# Global Distribution and Clinical Features of Pythiosis in Humans and Animals

**DOI:** 10.3390/jof8020182

**Published:** 2022-02-11

**Authors:** Hanna Yolanda, Theerapong Krajaejun

**Affiliations:** 1 Program in Translational Medicine, Faculty of Medicine, Ramathibodi Hospital, Mahidol University, Bangkok 10400, Thailand; hannayolanda15@gmail.com; 2 Department of Parasitology, School of Medicine and Health Sciences, Atma Jaya Catholic University of Indonesia, Jakarta 14440, Indonesia; 3 Department of Pathology, Faculty of Medicine, Ramathibodi Hospital, Mahidol University, Bangkok 10400, Thailand

**Keywords:** pythiosis, *Pythium insidiosum*, distribution, epidemiology, clinical feature

## Abstract

Pythiosis is a difficult-to-treat infectious disease caused by *Pythium insidiosum*. The condition is unfamiliar among healthcare workers. Manifestation of pythiosis is similar to other fungal infections, leading to misdiagnosis and delayed treatment. The geographical extent of pythiosis at a global scale is unclear. This study aimed to analyze the clinical information recorded in the scientific literature to comprehensively project epidemiological characteristics, clinical features, and future trends of pythiosis. From 1980 to 2021, 4203 cases of pythiosis in humans (*n* = 771; 18.3%) and animals (primarily horse, dog, and cow; *n* = 3432; 81.7%), with an average of 103 cases/year, were recruited. Pythiosis case reports significantly increased in the last decade. Pythiosis spanned 23 tropical, subtropical, and temperate countries worldwide. Some patients acquired pythiosis from a trip to an endemic country. Strikingly, 94.3% of human cases were in India and Thailand, while 79.2% of affected animals were in the U.S.A. and Brazil. Clinical features of pythiosis varied. Vascular and ocular pythiosis were only observed in humans, whereas cutaneous/subcutaneous and gastrointestinal infections were predominant in animals. Mortality depended on host species and clinical forms: for example, none in patients with ocular pythiosis, 0.7% in cows with a cutaneous lesion, 26.8% in humans with vascular disease, 86.4% in dogs with gastrointestinal pathology, and 100% in several animals with disseminated infection. In summary, this study reports up-to-date epidemiological and clinical features of pythiosis in humans and animals. It increases awareness of this life-threatening disease, as the illness or outbreak can exist in any country, not limited to the endemic areas.

## 1. Introduction

Pythiosis is an under-diagnosed and difficult-to-treat infectious disease [1]. High morbidity and mortality of pythiosis are critical healthcare problems. In the past, the disease was known as phycomycosis [2,3,4,5], bursattee [6], espundia [7], horse leeches [2,8], granular dermatitis [9], summer sore [9], and swamp cancer [10,11]. The etiologic agent is the filamentous eukaryotic microorganism called *Pythium insidiosum* [12], which taxonomically belongs to the Stramenopiles-Alveolata-Rhizaria supergroup [13]. Microscopic characteristics of *P. insidiosum* include broad hyphae (4–10 µm in diameter), perpendicular branching, coenocyte (in young hyphae), sparse septation (in an aged organism), and rounded hyphal tips [12]. The organism produces biflagellate zoospores (asexual reproductive stage) in a suitable aquatic environment [9,12,14] and requires a water plant for colonization as a part of its life cycle [15].

Presumptive pythiosis cases, clinically presented with granulomatous lesions, have been reported since the 19th century [6]. In 1901, the causative agent of this granulomatous disease was isolated and named “*Hyphomycosis destruens*” by de Haan and Hoogkamer [16]. In 1974, Austwick and Copland isolated a similar organism from horses with a granulomatous lesion [14]. They successfully induced the organism to produce biflagellate zoospores. Based on this unique morphological feature, they reported that the organism in question belongs to the genus *Pythium* [14]. Since then, an animal-isolated organism with the compatible microscopic characteristics described by Austwick (1974) has been referred to as a *Pythium* species [17,18,19,20]. In 1987, after an extensive microbiological characterization, de Cock et al. assigned such organism, isolated from animals and humans to the species level as “*Pythium insidiosum*” [12]. In the same year, Shipton also assigned the same organism as “*Pythium destruens*” [21,22], which is infrequently referred to in the literature.

Direct contact of an individual or animal to *P. insidiosum* (i.e., zoospores) could initiate an infection [15,23]. The clinical manifestation of pythiosis can be similar to other fungal infections [11,24], leading to misdiagnosis, delayed treatment, and poor prognosis. Some diagnostic modalities have been developed and used for diagnosing pythiosis, such as culture identification and zoospore induction [25,26,27,28], histological examination (i.e., immunostaining) [29,30,31,32,33,34], serological tests [i.e., immunodiffusion (ID), Western blot (WB), enzyme-linked immunosorbent assay (ELISA), hemagglutination (HA), and immunochromatography (ICT)] [35,36,37,38,39,40,41], molecular assays (i.e., PCR and sequence homology analysis) [42,43,44,45], and proteomic approaches [46,47]. *P. insidiosum* exhibits limited sensitivity to conventional antimicrobial agents [48,49]. Surgical intervention to remove an infected tissue is the primary treatment for pythiosis [50,51]. Immunotherapy using *P. insidiosum* antigens has been implemented to treat pythiosis, but its curative efficacy needs improvement [52].

Pythiosis has been found in several animal species, including horses [3,7,17,53], cattle [18], and dogs [19]. The first occurrence of pythiosis in humans was documented in 1985 [54,55]. In the past decades, new pythiosis cases have been increasingly reported in the literature [3,56,57,58,59,60,61]. The geographical extent and burden of pythiosis at a global scale are unknown. Here, we gathered and analyzed the clinical information of pythiosis recorded in the scientific literature. This study comprehensively reports up-to-date epidemiological characteristics, geographic distribution, clinical features, overall mortalities, and future trends of pythiosis. The obtained information will provide insights and promote awareness of pythiosis among healthcare personnel, leading to better preventive and therapeutic measures for this under-recognized and devastating disease.

## 2. Methods

The keywords “*Pythium insidiosum*”, “pythiosis”, “pitiose”, and “pitiosis” were used to search for human and animal patients with pythiosis deposited (up until December 2021) in several public databases, such as PubMed (https://pubmed.ncbi.nlm.nih.gov; accessed date: 19 January 2022), SciELO (https://scielo.org; 19 January 2022), and Google Scholar (https://scholar.google.com; 19 January 2022). In addition, we also searched for more pythiosis cases from available local journals/databases (some of which were written in non-English languages). Pythiosis cases (both humans and animals) and associated clinical data (i.e., publication year, number of cases, country of origins, host species, clinical manifestations, diagnosis, treatment, and clinical outcomes) were extracted and collected from the obtained literature. All collected cases must be diagnosed as “pythiosis” by at least one of the following diagnostic procedures: (i) microbiological methods (i.e., culture identification, zoospore induction, and microscopic examination); (ii) histological assessment (i.e., histological examination and staining); (iii) serological test (i.e., ID, WB, ELISA, HA, and ICT); (iv) molecular assay (i.e., PCR and sequence homology); and (v) proteomic approach (i.e., MALDI-TOF) [26,31,35,44,46,55]. Potentially duplicated or repeatedly reported pythiosis patients (including those that lacked information on animal species) were excluded from the study to prevent overestimating the overall case number.

Geographic locations of the reported pythiosis patients were marked on the world map using the Microsoft EXCEL software version 2111. Moreover, research articles reporting successful isolation of *P. insidiosum* from the environment were also collected, and the ecological niches of the organism were mapped together with the pythiosis case distribution. Frequencies of the reports and the case numbers were demonstrated as bar graphs according to publication years. The recruited cases (total number and percentage) were classified based on the countries of origin, clinical manifestations, affected host species, and clinical outcomes.

## 3. Results

### 3.1. Pythiosis Case Recruitment

**Figure 1:**
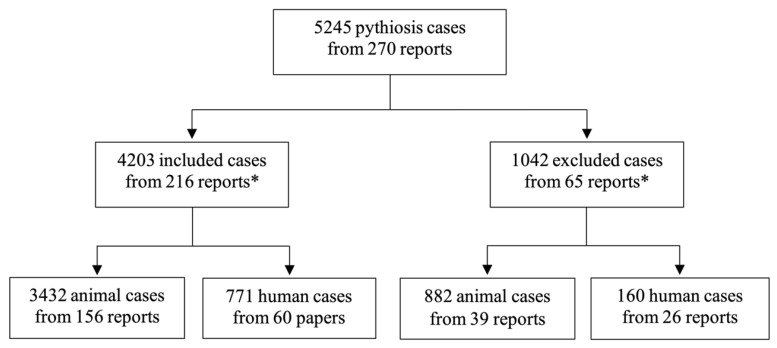
The number of included and excluded pythiosis cases from 270 reports published from 1980–2021. Clinical information of 1042 patients from 65 reports is excluded from the study due to considerably duplicated or repeated cases reported in other publications or lacking information on animal species. Eleven reports (as indicated by the asterisks) contain both included (*n* = 1830) and excluded (*n* = 133) cases of pythiosis. Four thousand two hundred and three pythiosis cases from 216 reports were recruited for epidemiological analyses.

By searching through the scientific literature databases (i.e., PubMed, SciELO, and Google Scholar) and some local journals (using the keywords “*Pythium insidiosum*”, “pythiosis”, “pitiose”, and “pitiosis”), a total of 270 articles reporting pythiosis cases were obtained (Supplementary Table S1). These reports were manually screened for pythiosis patients diagnosed by an established method (i.e., microbiological identification, histological assessment, serological test, molecular assay, or proteomic approach; see the Methods for more details). As a result, 5245 human and animal cases of pythiosis and their associated clinical data were recruited from the published articles (Figure 1; Supplementary Table S1). However, 1042 patients (882 animals and 160 humans) from 65 articles were excluded from the study because they were considerably duplicated cases. Finally, 4203 cases of pythiosis (3432 animals and 771 humans) reported in 216 articles were included for downstream epidemiological analyses (Figure 1; Supplementary Table S1).

### 3.2. Frequencies and Geographic Distribution of Pythiosis Cases

**Figure 2:**
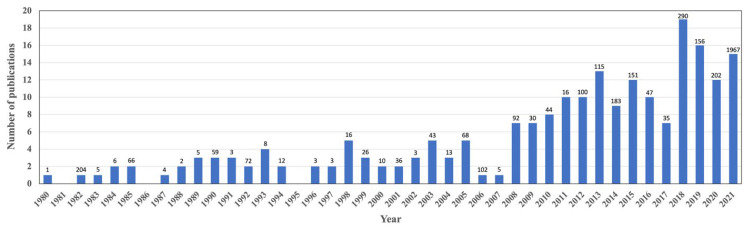
Distribution of 216 published articles reporting 4203 pythiosis cases in humans and animals during 1980–2021. The number above each bar graph indicates the total reported cases in each respective year.

**Figure 3:**
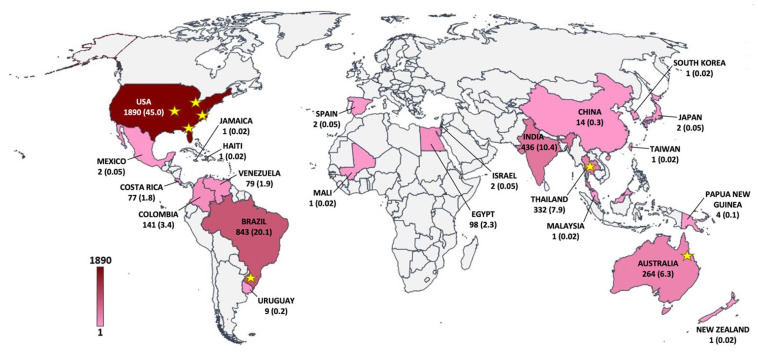
The world map shows the geographic distribution of pythiosis in humans and animals. The case numbers and percentages (in the parenthesis) are added to the countries where the patients acquired pythiosis. The color scale represents case density (ranging from 1 to 1890 cases). Stars indicate the areas or countries where *P. insidiosum* has been successfully isolated from the environment.

A total of 216 scientific articles reporting 4203 unique cases of pythiosis in animals (*n* = 3432; 81.7%) and humans (*n* = 771; 18.3%) were published between January 1980 and December 2021 (Figure 2). During these 41 years of publications, the average number of documented pythiosis patients was 103 cases/year. About half of the recruited articles (123 out of 216; 56.9%) containing clinical data of 3246 humans and animals with pythiosis (77.2% of all cases) were published in the last 10 years (2012–2021). The first report of the disease, so-called “pythiosis” (in horses), was in 1983 from Australia [4]. Afterward, pythiosis had been increasingly reported from 23 different countries throughout the world (Figure 3; Table 1). Most pythiosis cases (*n* = 4187; 99.6%) were reported from U.S.A. (*n* = 1890; 45.0%), followed by Brazil (*n* = 843; 20.1%), India (*n* = 436; 10.4%), Thailand (*n* = 332; 7.9%), Australia (*n* = 264; 6.3%), Colombia (*n* = 141; 3.4%), Egypt (*n* = 98; 2.3%), Venezuela (*n* = 79; 1.9%), Costa Rica (*n* = 77; 1.8%), China (*n* = 14; 0.3%), Uruguay (*n* = 9; 0.2%), and Papua New Guinea (*n* = 4; 0.1%). An additional 16 pythiosis cases (11 humans and 5 animals; 1–2 cases/country) were from Japan, Israel, Spain, Mexico, Malaysia, South Korea, Jamaica, Haiti, Mali, New Zealand, Taiwan, and an uncertain country (Brazil or Colombia) (Table 1). Among the patients mentioned earlier, five cases were diagnosed with “pythiosis” in their home countries (i.e., France, Spain, and the U.S.A.) after an international trip to Brazil, Colombia, Costa Rica, Israel, Spain, or Thailand (Table 2) [62,63,64,65,66,67]. These patients (who all had ocular pythiosis) were defined as imported cases and then mapped to the known country where they likely acquired the infection (Figure 3).

### 3.3. Ecological Distribution of P. insidiosum

Nine scientific articles described the successful isolations and molecular detections of *P. insidiosum* in water or soil samples from the environment in four countries (i.e., U.S.A., Brazil, Thailand, and Australia; as indicated by the stars in Figure 3) [4,68,69,70,71,72,73,74,75]. *P. insidiosum* was first isolated from swampy areas in Queensland, Australia [4]. The pathogen was also found to circulate in some Southeast and Midwest states of the U.S.A. (i.e., Kansas, Missouri, Ohio, Florida, and Virginia) [69,72,73,74]. Recovery of the organism was documented in the Rio Grande do Sul state of Southern Brazil [75]. In addition, three studies in Thailand demonstrated that *P. insidiosum* ubiquitously inhabits rice fields and water reservoirs in the Northern, Central, and Southern provinces [68,70,71].

### 3.4. Affected Animal Types

Pythiosis primarily affected vertebrates (*n* = 4199), such as humans, horses, dogs, cows, sheep, cats, donkeys, mules, camels, bears, birds, goat, tiger, and jaguar (Table 3). However, a few invertebrate species (i.e., shrimp, worms, and mosquito larvae; *n* = 4) can be infected by *P. insidiosum* [76,77,78]. Among all reported pythiosis cases, the frequently affected hosts included horses (*n* = 2312; 55.0%), followed by humans (*n* = 771; 18.3%), dogs (*n* = 664; 15.8%), cows (*n* = 292; 7.0%), sheep (*n* = 86; 2.1%), and cats (*n* = 41; 1.0%) (Table 3). The other affected vertebrates (i.e., donkeys, mules, camels, bears, birds, goat, tiger, and jaguar; *n* = 33) altogether accounted for 0.8% of all reported cases [78,79,80,81,82,83,84,85,86,87,88,89]. Pythiosis in animals has been mainly reported from the U.S.A. (*n* = 1876; 54.7% of all animal cases), Brazil (*n* = 842; 24.5%), Australia (*n* = 261; 7.6%), Colombia (*n* = 141; 4.1%), Egypt (*n* = 98; 2.9%), Venezuela (*n* = 79; 2.3%), and Costa Rica (*n* = 76; 2.2%), whereas the disease in humans has been primarily discovered in India (*n* = 434; 56.3% of all human cases) and Thailand (*n* = 293; 38.0%) (Table 1).

### 3.5. Clinical Features of Pythiosis in Humans and Animals

Clinical manifestations of pythiosis can be classified into cutaneous/subcutaneous, ocular, vascular, gastrointestinal, pulmonary, prostatic, and disseminated/systemic pythiosis (Table 3). Clinical characteristics of cutaneous/subcutaneous pythiosis included ulcerative granulomatous lesion, cellulitis, and subcutaneous lumps, which can be observed at various anatomical parts, such as the head and neck (covering face, periorbital area, nose, lip, and oral cavity), body (i.e., chest and abdomen), perineum (including genitalia and anus), and limb (underneath the muscle and joint could be affected) [82,90,91,92,93,94,95,96,97,98,99,100,101]. An ocular lesion was limited within an eye globe. Ocular pythiosis cases are usually present with corneal ulcer or keratitis (also called *Pythium* keratitis) [65,102,103,104,105,106]. Patients with vascular pythiosis often came with an infection of a medium- to large-size artery (arteritis), leading to inflammation of the adjacent tissue, arterial wall thickening, aneurysm, vascular lumen occlusion (such as by thrombosis), and gangrenous tissue [107,108,109,110,111]. Gastrointestinal pythiosis cases can exhibit a pathological lesion (i.e., mass and ulcer) in the esophagus, stomach, or intestine, in which the disease could involve the liver, spleen, pancreas, omentum, and mesentery [85,112,113,114,115,116,117]. Pulmonary pythiosis comprises the infection of the lung and trachea [53,88,118,119]. A *P. insidiosum* infection of the prostate gland was referred to as prostatic pythiosis [120]. Disseminated (or systemic) pythiosis can be defined as the *P. insidiosum* infection of multiple organs, such as the artery, skin, gastrointestinal tract, bone, lung, brain, and other internal organs [55,90,121,122,123,124,125,126,127,128,129,130,131,132].

Clinical manifestations of pythiosis in humans and animals were different to some extent (Table 3). Ocular and vascular pythiosis were observed only in humans, while cutaneous/subcutaneous and gastrointestinal pythiosis were much more prevalent in animals. Disseminated pythiosis was uncommon in both humans and animals (0.9% of all clinical forms). In humans (*n* = 771), the majority of the patients were presented with ocular pythiosis (*n* = 572; 74.2%), followed by vascular (*n* = 169; 21.9%), cutaneous/subcutaneous (*n* = 19; 2.5%), and disseminated (*n* = 11; 1.4%) pythiosis (Table 3). Most ocular pythiosis cases (*n* = 547; 95.6%) were reported from India (*n* = 433; 75.7%) and Thailand (*n* = 114; 19.9%), while the rest (*n* = 25; 4.4%) were from China (*n* = 14; 2.4%), Israel (*n* = 2; 0.3%), Spain (*n* = 2), Malaysia (*n* = 1; 0.2%), Costa Rica (*n* = 1), Japan (*n* = 1), Haiti (*n* = 1), Australia (*n* = 1), New Zealand (*n* = 1), and Brazil or Colombia (*n* = 1). Vascular pythiosis cases were almost exclusively discovered in Thailand (*n* = 168; 99.4%) [55,60,107,110,111,133,134,135,136], while only one case was discovered in Jamaica [137].

Animals with pythiosis predominantly manifested with a cutaneous/subcutaneous infection at the face, limb, thorax, or abdomen (*n* = 3082; 89.8% of all affected animals; Table 3) [138,139,140,141,142,143,144,145,146,147,148,149]. To a much lesser extent, some affected animals (*n* = 350; 10.2%) came with clinical manifestations associated with gastrointestinal (*n* = 249; 7.3%), disseminated (*n* = 25; 0.7%), pulmonary (*n* = 4; 0.1%), or other organ (*n* = 5; 0.1%) infection (Table 3). Gastrointestinal pythiosis (*n* = 249) was mostly observed in dogs (*n* = 235; 94.4%), but rarely detected in other animal species, such as horses (*n* = 5; 2.0%) [150,151,152,153,154], cats (*n* = 4; 1.6%) [155,156,157], sheep (*n* = 2; 0.8%) [158], camels (*n* = 1; 0.4%) [159], ostrich (*n* = 1) [85], and tiger (*n* = 1) [87] (Table 3). Disseminated pythiosis in animals usually began with a cutaneous/subcutaneous infection and progressed to the bone, liver, lung, or other organs [126,127,128,129,130,131,132,160].

### 3.6. Clinical Outcomes of Pythiosis

Regardless of hospital course, case severity, and treatment, clinical outcome information (dead vs. survived) was available from 1497 (1100 animals and 397 humans; 35.6%) out of all 4203 recruited pythiosis patients (Table 4). The mortality rate of pythiosis was 33.5% in animals, 12.8% in humans, and 28.1% overall. Among the frequently affected hosts (overall case number > 50), dogs possessed the highest mortality rate (83.9%), respectively followed by sheep (71.7%), equines (including horses, mules, and donkeys; 25.3%), humans (12.8%), and cows (0.7%). Cutaneous/subcutaneous and disseminated pythiosis were found in humans and animals. These clinical forms exhibited various mortality rates, depending on the affected host species: for example, 0.7% in cows with cutaneous/subcutaneous pythiosis, 70.8% in sheep with cutaneous/subcutaneous disease, 88.9% in humans with disseminated condition, and 100.0% in dogs with disseminated infection (Table 4). Gastrointestinal infection was the most common pythiosis in dogs and showed markedly high mortality (86.4%). Pulmonary pythiosis was rarely but primarily found in animals (i.e., horses, dogs, and jaguars; *n* = 4) and associated with an extremely high chance (100.0%) of a fatal outcome.

In humans, no pythiosis patient died from a confined ocular infection, although many lost an infected eye as a part of the treatment to control the disease progression. In contrast, humans with disseminated pythiosis posted the highest mortality rate (88.9%), followed by those with vascular (26.8%) and cutaneous/subcutaneous (13.3%) diseases. It should be noted that there was a human patient, defined as a disseminated pythiosis case, who was presented with an ocular infection that progressed to the cavernous sinus, leading to a fatal cerebrovascular attack [122]. When only considering the vascular, cutaneous/subcutaneous, and disseminated infections (*n* = 177), the mortality rate of human patients with non-ocular pythiosis was 28.8%. Morbidity information was noticed in some human patients with vascular and ocular pythiosis. For example, 92 out of 106 vascular patients (86.8%) underwent an amputation of the affected limb. Approximately two-thirds of the amputated cases (*n* = 63; 68.5%) survived the disease, while the rest (*n* = 29; 31.5%) passed away as the infection recurred or progressed. Regarding the human patients with ocular pythiosis, 112 out of 121 cases (92.6%) required surgical intervention, such as keratoplasty (*n* = 63; 56.3%) or eye removal (i.e., evisceration, enucleation, and exenteration; *n* = 49; 43.7%) to cure the *P. insidiosum* infection.

## 4. Discussion

Pythiosis is recognized as an infectious disease with high morbidity and mortality. The disease is unfamiliar among healthcare workers partly because it is relatively rare compared with other infectious conditions. As presented here, thousands of pythiosis patients reported in 23 countries (Figure 3; Table 1) could be just a portion of what existed. The exact number of cases and geographic extent of pythiosis is unknown. However, increased reports of pythiosis in many countries worldwide (Figure 2 and Figure 3) indicate that the disease is widespread and should be a public health concern, as an infection outbreak is possible [161,162,163,164,165]. The countries such as Thailand, India, Northeastern Australia, Brazil, Colombia, Venezuela, Uruguay, Costa Rica, and the Southern/Southeastern states of the U.S.A., where the majority of all pythiosis cases were located, are in the tropical and subtropical regions (Figure 3; Table 1). This observation is in line with the fact that an optimal growth temperature of *P. insidiosum* is between 28 and 37 °C [12,25]. The organism can still grow at temperatures up to 45 °C [12]. However, temperatures as low as 8 °C can inhibit the pathogen growth [25]. Thus, *P. insidiosum* tends to circulate in a relatively warm environment of such tropical and subtropical countries, and in some of which (i.e., Thailand, Brazil, U.S.A., and Australia), the organism was successfully isolated from water and soil (Figure 3) [4,68,69,70,71,72,73,74,75]. Nevertheless, to a much lesser extent, several pythiosis cases can be found in temperate areas, such as New Zealand [166], Spain [67,167], South Korea [168], and the state of Wisconsin, U.S.A. [56].

After excluding all possible duplicated pythiosis patients (*n* = 1042) (Figure 1), a total of 4203 unique pythiosis cases have been documented in 216 scientific articles over the past 41 years (1980–2021), with an average of 103 patients annually (Figure 2). Some cases were discovered for years before publication. For example, an ocular pythiosis patient was found 13 years before the clinical detail was reported in 1997 [166], and animal patients were recorded during 2000–2005 and 2016–2020 before the case collection was published in 2021 [169]. Strikingly, close to 80% of all recruited pythiosis patients were documented in the literature during the last decade (Figure 2). Thus, discovering a new pythiosis case tends to increase over time, and is evidenced by a higher frequency of publications and reported cases. This observation could result from more healthcare personnel recognizing pythiosis, and diagnostic tools becoming publicly available.

Another interesting observation regarding the global distribution and frequency of pythiosis is the infected host species covering humans and various animals (Table 3). Of all pythiosis cases, horses (accounted for 55.0%), humans (18.3%), dogs (15.8%), and cows (7.0%) formed the primarily affected hosts. A small portion (0.1%) of the infected animals were invertebrates (i.e., mosquito larva, shrimp, and worms), suggesting *P. insidiosum* infects a broad host range and might play an interactive role in the ecosystem. Most animal pythiosis cases (*n* = 2718; 79.2% of all affected animals) were in the U.S.A. and Brazil, which are the countries in the Americas (Figure 3; Table 1). In contrast, almost all human pythiosis cases (*n* = 727; 94.3% of all affected humans) had been reported from two Asian countries: India and Thailand (Figure 3; Table 1). Different genotypes of *P. insidiosum* in different continents were observed; for example, the clade-I genotype is almost exclusively found in the Americas, and the clade-II and -III genotypes are commonly identified in Asia [170,171]. Thus, the pathogen genotypes may involve the selection of a preferable host (i.e., animal vs. human).

A history of contacting fresh water before the clinical symptoms of pythiosis was often obtained from the patients or animal owners [87,92,117,135,138,158,164,165,172]. *P. insidiosum* inhabits swampy areas, including rice fields, rivers, cannel, and water reservoirs [4,68,69,70,71,72,73,74,75]. Direct exposure to *P. insidiosum*-contaminated water could initiate the infection [15]. A motile zoospore (an infective unit of *P. insidiosum*) can attach and penetrate a host tissue causing pythiosis [173,174]. Routine activity and hygiene of an individual may contribute to a different infection rate. Horses, dogs, and cows are the most affected species that could expose the *P. insidiosum* habitat (i.e., a swampy area) more frequently than the other domestic animals. In Thailand, the majority of the human patients were farmers, who had an increased risk of acquiring pythiosis by regularly working in a rice field, likely without proper protection (i.e., wearing glasses, gloves, and boots) [42,55,60,107,110,123,133,134,175]. Regarding personal hygiene, wearing a contaminated contact lens and direct eye exposure to water leads to an increased chance of acquiring the *P. insidiosum* infection [42,55,62,66,67,122,162,176,177,178].

As mentioned above, Thailand and India accounted for the highest prevalence of pythiosis in humans (Table 2). Surges of human cases (all had ocular pythiosis) were published by several Indian medical centers during the last six years (2015–2021; Figure 2) [50,59,105,122,179,180,181,182,183,184,185,186,187,188], leading India to report the highest case number of human pythiosis so far. Surprisingly, no vascular pythiosis was diagnosed in India, while almost all patients with this disease type (99.4%) were detected in Thailand. A host factor might involve this phenomenon. For example, the vascular pythiosis patients also had an underlying hematological condition, mostly thalassemia [42,55]. Thalassemia is highly prevalent in various regions of the world, particularly Thailand and other Southeast Asian countries [189]. It remains unknown how thalassemia is associated with pythiosis, perhaps due to thalassemia-mediated immune impairment [189]. Some patients living in a country with no previous report of pythiosis acquired the disease from an international trip to another nation (Table 2). Thus, pythiosis might not be uncommon and can be discovered anywhere, and not limited to the endemic countries. Awareness of the illness by healthcare personnel and availability of a diagnostic tool in clinical laboratories are critical for detecting new pythiosis cases.

Surgery is the primary treatment for pythiosis. Additional therapies included antimicrobial drugs (mostly ineffective) [48] and immunotherapy (limited availability and efficacy) [52]. The mortality of pythiosis depended on animal species and clinical forms (i.e., vascular, ocular, cutaneous/subcutaneous, gastrointestinal, pulmonary, and disseminated infections; Table 4), which may involve microbial pathogenicity and host susceptibility or immune status. Based on the available information about clinical outcomes (dead vs. survived), the overall mortality of pythiosis in animals was more remarkable than in humans (33.5% vs. 12.8%) (Table 4). This observation may be in part due to the typical clinical forms of pythiosis and their associated mortality rates in humans and animals, which were different: the cutaneous/subcutaneous infection in animals (90% of all animal cases; up to 71% mortality depending on host species) and the ocular disease in humans (74% of all human cases; no mortality outcome) (Table 3 and Table 4). In animals, dogs exhibited the highest overall mortality rates (84%) and were markedly higher than the other affected species because gastrointestinal pythiosis, which posted a very high fatal outcome (up to 100% in some species: Table 4), was way more prevalent in canine cases (Table 3). In contrast, cows with cutaneous/subcutaneous pythiosis showed significantly lower mortality rates (<1%) than most other animals with the same clinical form (Table 4). Regardless of host types, disseminated and pulmonary pythiosis, although relatively rare, usually led to a fatal outcome. Like other animals, different pythiosis forms in human patients contributed varied mortality rates, ranging from 0% in ocular disease to 89% in disseminated infection (Table 4). Pythiosis also had high morbidity. For example, most patients with vascular (87%) and ocular (93%) infection received a surgical intervention (i.e., leg amputation and cornea replacement) to control the disease. Post-surgically, despite intensive care provided, a significant number of these vascular cases succumbed to pythiosis (32%), and a remarkable number of such ocular patients lost their infected eyes (44%).

In conclusion, the pythiosis cases were collected from the scientific literature published over the past 41 years (1980–2021). Reports of pythiosis patients have increased, especially in the last decade. In total, 4203 cases of pythiosis in humans (*n* = 771) and various animals (primarily horses, dogs, and cows; *n* = 3432), with an average of 103 cases/year, were recruited for epidemiological analysis. Pythiosis spanned across 23 tropical, subtropical, and temperate countries worldwide. Some patients have acquired pythiosis from an international trip to another country. Most human cases were in India and Thailand, while most animal cases were in the U.S.A and Brazil. Clinical features of pythiosis varied. Vascular and ocular pythiosis were only observed in humans, whereas cutaneous/subcutaneous and gastrointestinal infections were predominantly observed in animals. Mortality depended on host species and clinical forms, ranging from none in human patients with ocular pythiosis to 100% in animals with disseminated infection. This study comprehensively projected up-to-date epidemiological and clinical features of pythiosis in humans and animals globally. It increased awareness of this life-threatening disease, as the illness and outbreak could exist in many countries, not limited to only the endemic areas.

## Figures and Tables

**Table 1:** Distribution of human and animal pythiosis in 23 countries.

Countries	Humans	Animals					Overall Cases (%)
		Horses	Dogs	Cows	Other ^a^	All	
U.S.A.	14	1184	623	25	44	1876	1890 (44.97)
Brazil	1	577	29	132	104	842	843 (20.07)
India	434	-^b^	-	-	2	2	436 (10.37)
Thailand	293	32	7	-	-	39	332 (7.90)
Australia	3	259	2	-	-	261	264 (6.28)
Colombia	-	56	-	72	13	141	141 (3.35)
Egypt	-	98	-	-	-	98	98 (2.33)
Venezuela	-	15	1	63	-	79	79 (1.89)
Costa Rica	1	76	-	-	-	76	77 (1.83)
China	14	-	-	-	-	-	14 (0.33)
Uruguay	-	9	-	-	-	9	9 (0.22)
Papua New Guinea	-	4	-	-	-	4	4 (0.10)
Japan	1	1	-	-	-	1	2 (0.05)
Israel	2	-	-	-	-	-	2 (0.05)
Spain	2	-	-	-	-	-	2 (0.05)
Mexico	1	1	-	-	-	1	2 (0.05)
Malaysia	1	-	-	-	-	-	1 (0.02)
South Korea	-	-	1	-	-	1	1 (0.02)
Jamaica	1	-	-	-	-	-	1 (0.02)
Haiti	1	-	-	-	-	-	1 (0.02)
Mali	-	-	1	-	-	1	1 (0.02)
New Zealand	1	-	-	-	-	-	1 (0.02)
Taiwan	-	-	-	-	1	1	1 (0.02)
Uncertain ^c^	1	-	-	-	-	-	1 (0.02)
Total cases (%)	771 (18.34)	2312 (55.01)	664 (15.80)	292 (6.95)	164 (3.90)	3432 (81.66)	4203 (100.00)

^a^ Other animals include cat, mule, donkey, sheep, camel, bird, goat, tiger, jaguar, bear, worm, mosquito larva, and shrimp. ^b^ No case is reported. ^c^ The country (Brazil or Colombia) where the patient acquired pythiosis is uncertain.

**Table 2:** Five imported cases of pythiosis who acquire the *P. insidiosum* infection outside their home countries.

Case	Authors	Year of Publication	Home Country ^a^	Visited Country ^b^	Reference
1	Tanhehco et al.	2011	U.S.A.	Israel	[62]
2	Lelievre et al.	2015	France	Thailand	[64]
3	Ros Castellar et al.	2017	Spain	Brazil and Colombia ^c^	[65]
4	Neufeld et al.	2018	U.S.A.	Costa Rica	[63,66]
5	Bernheim et al.	2019	France	Spain	[67]

^a^ Home country is where the patients live and receive the diagnosis and treatment of pythiosis. ^b^ Visited country refers to where the patients visit and acquire the *P. insidiosum* infection. ^c^ The country where a patient acquires pythiosis is uncertain.

**Table 3:** The number of pythiosis cases based on host types and clinical forms.

Host Types	Clinical Forms						Total Cases (%)
	Vascular Pythiosis	Ocular Pythiosis	Cutaneous/Subcutaneous Pythiosis	Gastrointestinal Pythiosis	Disseminated Pythiosis	Others or Unknown ^a^	
Humans	169	572	19	-^b^	11	-	771 (18.34)
Animals (overall)	-	-	3082	249	25	76	3432 (81.66)
Horses	-	-	2267	5	16	24	2312 (55.01)
Dogs	-	-	421	235	4	4	664 (15.80)
Cows	-	-	274	-	-	18	292 (6.95)
Sheep	-	-	81	2	3	-	86 (2.05)
Cats	-	-	11	4	1	25	41 (0.98)
Donkeys	-	-	15	-	-	-	15 (0.36)
Mules	-	-	7	-	-	-	7 (0.16)
Camels	-	-	2	1	1	-	4 (0.10)
Bears	-	-	2	-	-	-	2 (0.05)
Birds	-	-	1	1	-	-	2 (0.05)
Mosquito larvae	-	-	-	-	-	2	2 (0.05)
Shrimp	-	-	-	-	-	1	1 (0.02)
Worm	-	-	-	-	-	1	1 (0.02)
Goat	-	-	1	-	-	-	1 (0.02)
Tiger	-	-	-	1	-	-	1 (0.02)
Jaguar	-	-	-	-	-	1	1 (0.02)
All humans and animals (%)	169 (4.02)	572 (13.61)	3101 (73.78)	249 (5.92)	36 (0.86)	76 (1.81)	4203 (100.00)

^a^ Pythiosis of the lung, prostate gland, exoskeleton, or unknown site. ^b^ No case is reported

**Table 4:** The mortality rate of pythiosis is classified according to host types (i.e., humans and other animals) and clinical forms (i.e., vascular, ocular, cutaneous/subcutaneous, gastrointestinal, pulmonary, and disseminated infections).

Host Type and Clinical Form	Number of Pythiosis Cases with Clinical Outcome	Number of Mortal Cases	Mortality Rate (%)
**1. Humans (overall)**	**397**	**51**	**12.8**
1.1 Vascular pythiosis	153	41	26.8
1.2 Ocular pythiosis	220	-^a^	0.0
1.3 Cutaneous/subcutaneous pythiosis	15	2	13.3
1.4 Disseminated pythiosis	9	8	88.9
**2. Animals (overall)**	**1100**	**369**	**33.5**
**2.1 Equines (horses, mules, and donkeys)**	**735**	**186**	**25.3**
2.1.1 Cutaneous/subcutaneous pythiosis	713	167	23.4
2.1.2 Gastrointestinal pythiosis	4	2	50.0
2.1.3 Pulmonary pythiosis	2	2	100.0
2.1.4 Disseminated pythiosis	16	15	93.8
**2.2 Dogs**	**155**	**130**	**83.9**
2.2.1 Cutaneous/subcutaneous pythiosis	31	22	71.0
2.2.2 Gastrointestinal pythiosis	118	102	86.4
2.2.3 Pulmonary pythiosis	1	1	100.0
2.2.4 Prostatic pythiosis	1	1	100.0
2.2.5 Disseminated pythiosis	4	4	100.0
**2.3 Sheep**	**53**	**38**	**71.7**
2.3.1 Cutaneous/subcutaneous pythiosis	48	34	70.8
2.3.2 Gastrointestinal pythiosis	2	2	100.0
2.3.3 Disseminated pythiosis	3	2	66.7
**2.4 Cats**	**12**	**7**	**58.3**
2.4.1 Cutaneous/subcutaneous pythiosis	8	5	62.5
2.4.2 Gastrointestinal pythiosis	3	2	66.7
2.4.3 Disseminated pythiosis	1	-	0.0
**2.5 Camels**	**4**	**3**	**75.0**
2.5.1 Cutaneous/subcutaneous pythiosis	2	1	50.0
2.5.2 Gastrointestinal pythiosis	1	1	100.0
2.5.3 Disseminated pythiosis	1	1	100.0
**2.6 Birds**	**2**	**1**	**50.0**
2.6.1 Cutaneous/subcutaneous pythiosis	1	1	100.0
2.6.2 Gastrointestinal pythiosis	1	-	0.0
**2.7 Cows (Cutaneous/subcutaneous pythiosis)**	**135**	**1**	**0.7**
**2.8 Goat (Cutaneous/subcutaneous pythiosis)**	**1**	**-**	**0.0**
**2.9 Shrimp (Exoskeleton infection)**	**1**	**1**	**100.0**
**2.10 Jaguar (Pulmonary pythiosis)**	**1**	**1**	**100.0**
**2.11 Tiger (Gastrointestinal pythiosis)**	**1**	**1**	**100.0**
**3. All humans and animals**	**1497**	**420**	**28.1**

^a^ No mortal case is reported.

## Data Availability

Not applicable.

## Supplementary Materials

The following supporting information can be downloaded at: https://www.mdpi.com/article/10.3390/jof8020182/s1, Table S1: Included and excluded publications (*n* = 270) for analyzing epidemiological and clinical features of pythiosis in humans and animals. Eleven reports contain both included (*n* = 1830) and excluded (*n* = 133) cases of pythiosis.
