# Supplementary material for: Global Distribution and Clinical Features of Pythiosis in Humans and Animals
Source: J Fungi (Basel). 2022 Feb 11;8(2):182. doi: 10.3390/jof8020182 (PMC8879638; doi:10.3390/jof8020182)
Supplement: Supplementary file 1 [file jof-08-00182-s001.zip › jof-1593468-supplementary.pdf]

Journal of Fungi

MDPI

Supplementary Table S1. Included and excluded publications (n = 270) for analyzing epidemiological and clinical features of pythiosis in humans and animals. Eleven reports contain both included (n = 1830) and excluded (n = 133) cases of pythiosis. All publications are listed in the table footnote.

Countries	Included Articles (n = 216)		Excluded Articles (n = 65)	
	Humans (771 Cases)	Animals (3432 Cases)	Human (160 Cases)	Animals (882 Cases)
USA	1–9	10–58	-	56,59–65
Brazil	66	67–136	137	71,74,76,77,96,97,136,138–155
India	156–169	65,170	161,171–173	-
Thailand	174–191	192–195	196–215	192
Australia	216,217	218–221	-	222
Colombia	-	223–229	-	230,231
Egypt	-	232–236	-	-
Venezuela	-	237–240	-	241
Costa Rica	242	243–245	246	247
China	248–251	-	-	-
Uruguay	-	252–254	-	-
Papua New Guinea	-	255	-	-
Japan	256	257	-	-
Israel	258,259	-	-	-
Spain	260,261	-	-	-
Mexico	262	263	-	-
Malaysia	264	-	-	-
South Korea	-	265	-	-
Jamaica	266	-	-	-
Haiti	267	-	-	-
Mali	-	268	-	-
New Zealand	269	-	-	-
Taiwan	-	121	-	-
Uncertain	270	-	-	-

Footnotes: List of the published articles.

1. Hoffman, M.A.; Cornish, N.E.; Simonsen, K.A. A Painful Thigh Lesion in an Immunocompromised 11-Year-Old Boy. *Pediatr. Infect. Dis. J.* **2011**, *30*, 1011–1017.
2. Schloemer, N.J.; Lincoln, A.H.; Mikhailov, T.A.; Collins, C.L.; Di Rocco, J.R.; Kehl, S.C.; Chusid, M.J. Fatal Disseminated Pythium Insidiosum Infection in a Child with Diamond-Blackfan Anemia. *Infect. Dis. Clin. Pract.* **2013**, *21*, e24–e26.
3. Heath, J.A.; Kiehn, T.E.; Brown, A.E.; LaQuaglia, M.P.; Steinherz, L.J.; Bearman, G.; Wong, M.; Steinherz, P.G. Pythium Insidiosum Pleuropericarditis Complicating Pneumonia in a Child with Leukemia. *Clin. Infect. Dis.* **2002**, *35*, e60–e64.
4. Salipante, S.J.; Hoogestraat, D.R.; SenGupta, D.J.; Murphey, D.; Panayides, K.; Hamilton, E.; Castañeda-Sánchez, I.; Kennedy, J.; Monsaas, P.W.; Mendoza, L.; et al. Molecular Diagnosis of Subcutaneous Pythium Insidiosum Infection by Use of PCR Screening and DNA Sequencing. *J. Clin. Microbiol.* **2012**, *50*, 1480–1483.
5. Hilton, R.E.; Tepedino, K.; Glenn, C.J.; Merkel, K.L. Swamp Cancer: A Case of Human Pythiosis and Review of the Literature. *Br. J. Dermatol.* **2016**, *175*, 394–397.
6. Shenep, J.L.; English, B.K.; Kaufman, L.; Pearson, T.A.; Thompson, J.W.; Kaufman, R.A.; Frisch, G.; Rinaldi, M.G. Successful Medical Therapy for Deeply Invasive Facial Infection Due to Pythium Insidiosum in a Child. *Clin. Infect. Dis.* **1998**, *27*, 1388–1393.

7. Kirzhner, M.; Arnold, S.R.; Lyle, C.; Mendoza, L.L.; Fleming, J.C. Pythium Insidiosum: A Rare Necrotizing Orbital and Facial Infection. *J. Pediatr. Infect. Dis. Soc.* **2014**, *4*, e10–e13.
8. Rinaldi, M.G.; Seidenfeld, S.M.; Fotherbell, A.M.; McGough, D.A. Pythium Insidiosum Causes Severe Disease in a Healthy Boy. *Mycol Obs.* **1989**, *9*, 7–8.
9. Mendoza, L.; Prasla, S.H.; Ajello, L. Orbital Pythiosis: A Non-Fungal Disease Mimicking Orbital Mycotic Infections, with a Retrospective Review of the Literature. *Mycoses* **2004**, *47*, 14–23.
10. Oldenhoff, W.; Grooters, A.; Pinkerton, M.E.; Knorr, J.; Trepanier, L. Cutaneous Pythiosis in Two Dogs from Wisconsin, USA. *Vet. Dermatol.* **2014**, *25*, 52–e21.
11. Thieman, K.M.; Kirkby, K.A.; Flynn-Lurie, A.; Grooters, A.M.; Bacon, N.J. Diagnosis and Treatment of Truncal Cutaneous Pythiosis in a Dog. *J. Am. Vet. Med. Assoc.* **2011**, *239*, 1232–1235.
12. Mendoza, L.; Mandy, W.; Glass, R. An Improved Pythium Insidiosum-Vaccine Formulation with Enhanced Immunotherapeutic Properties in Horses and Dogs with Pythiosis. *Vaccine* **2003**, *21*, 2797–2804.
13. Hensel, P.; Greene, C.E.; Medleau, L.; Latimer, K.S.; Mendoza, L. Immunotherapy for Treatment of Multicentric Cutaneous Pythiosis in a Dog. *J. Am. Vet. Med. Assoc.* **2003**, *223*, 215–218.
14. Dykstra, M.J.; Sharp, N.J.H.; Olivry, T.; Hillier, A.; Murphy, K.M.; Kaufman, L.; Kunkle, G.A.; Pucheu-Haston, C. A Description of Cutaneous-Subcutaneous Pythiosis in Fifteen Dogs. *Med. Mycol.* **1999**, *37*, 427–433.
15. Hnilica, K. Difficult Dermatologic Diagnosis. Pythiosis. *J. Am. Vet. Med. Assoc.* **1998**, *212*, 1192–1193.
16. Bentinck-Smith, J.; Padhye, A.A.; Maslin, W.R.; Hamilton, C.; McDonald, R.K.; Woody, B.J. Canine Pythiosis-Isolation and Identification of Pythium Insidiosum. *J. Vet. Diagn. Invest.* **1989**, *1*, 295–298.
17. Howerth, E.W.; Brown, C.C.; Crowder, C. Subcutaneous Pythiosis in a Dog. *J. Vet. Diagn. Invest.* **1989**, *1*, 81–83.
18. Cridge, H.; Hughes, S.M.; Langston, V.C.; Mackin, A.J. Mefenoxam, Itraconazole, and Terbinafine Combination Therapy for Management of Pythiosis in Dogs (Six Cases). *J. Am. Anim. Hosp. Assoc.* **2020**, *56*, 307.
19. Gaddis, K.L.; Brinkman, E.L. What Is Your Diagnosis? *J Am Vet Med A* **2017**, *250*, 1231–1233.
20. Thomas, R.C.; Lewis, D.T. Pythiosis in Dogs and Cats. *Compend. Contin. Educ. Pract. Vet. USA* **1998**, *20*, 63–72.
21. Reagan, K.L.; Marks, S.L.; Pesavento, P.A.; Maggiore, A.D.; Zhu, B.Y.; Grooters, A.M. Successful Management of 3 Dogs with Colonic Pythiosis Using Itraconzaole, Terbinafine, and Prednisone. *J. Vet. Intern. Med.* **2019**, *33*, 1434–1439.
22. Parambeth, J.C.; Lawhon, S.D.; Mansell, J.; Wu, J.; Clark, S.D.; Sutton, D.; Gibas, C.; Wiederhold, N.P.; Myers, A.N.; Johnson, M.C.; et al. Gastrointestinal Pythiosis with Concurrent Presumptive Gastrointestinal Basidiobolomycosis in a Boxer Dog. *Vet. Clin. Pathol.* **2019**, *48*, 83–88.
23. Dycus, D.L.; Fisher, C.; Butler, R. Surgical and Medical Treatment of Pyloric and Duodenal Pythiosis in a Dog. *J. Am. Anim. Hosp. Assoc.* **2015**, *51*, 385–391.
24. Aeffner, F.; Hall, M.J.; Pressler, B.M.; Townsend, K.L.; Papenfuss, T.L. Pathology in Practice. *J. Am. Vet. Med. Assoc.* **2015**, *246*, 511–513.
25. Schmiedt, C.W.; Stratton-Phelps, M.; Torres, B.T.; Bell, D.; Uhl, E.W.; Zimmerman, S.; Epstein, J.; Cornell, K.K. Treatment of Intestinal Pythiosis in a Dog with a Combination of Marginal Excision, Chemotherapy, and Immunotherapy. *J. Am. Vet. Med. Assoc.* **2012**, *241*, 358–363.
26. Connolly, S.L.; Frank, C.; Thompson, C.A.; Van Alstine, W.G.; Gelb, H.; Heng, H.G.; Klosterman, E.; Kiupel, M.; Grooters, A.M. Dual Infection with Pythium Insidiosum and Blastomyces Dermatitidis in a Dog. *Vet. Clin. Pathol.* **2012**, *41*, 419–423.
27. Hummel, J.; Grooters, A.; Davidson, G.; Jennings, S.; Nicklas, J.; Birkenheuer, A. Successful Management of Gastrointestinal Pythiosis in a Dog Using Itraconazole, Terbinafine, and Mefenoxam. *Med. Mycol.* **2011**, *49*, 539–542.
28. Berryessa, N.; Marks, S.; Pesavento, P.; Krasnansky, T.; Yoshimoto, S.; Johnson, E.; Grooters, A.M. Gastrointestinal Pythiosis in 10 Dogs from California. *J. Vet. Intern. Med.* **2008**, *22*, 1065–1069.

29. LeBlanc, C.J.; Echandi, R.L.; Moore, R.R.; Souza, C.; Grooters, A.M. Hypercalcemia Associated with Gastric Pythiosis in a Dog. *Vet. Clin. Pathol.* **2008**, *37*, 115–120.
30. Liljebjelke, K.A.; Abramson, C.; Brockus, C.; Greene, C.E. Duodenal Obstruction Caused by Infection with Pythium Insidiosum in a 12-Week-Old Puppy. *J. Am. Vet. Med. Assoc.* **2002**, *220*, 1188–1191.
31. Helman, R.G.; Oliver, J. Pythiosis of the Digestive Tract in Dogs from Oklahoma. *J. Am. Anim. Hosp. Assoc.* **1999**, *35*, 111–114.
32. Graham, J.P.; Newell, S.M.; Roberts, G.D.; Lester, N.V. Ultrasonographic Features of Canine Gastrointestinal Pythiosis. *Vet. Radiol. Ultrasound* **2000**, *41*, 273–277.
33. Patton, C.S.; Hake, R.; Newton, J.; Toal, R.L. Esophagitis Due to Pythium Insidiosum Infection in Two Dogs. *J. Vet. Intern. Med.* **1996**, *10*, 139–142.
34. Fischer, J.R.; Pace, L.W.; Turk, J.R.; Kreeger, J.M.; Miller, M.A.; Gosser, H.S. Gastrointestinal Pythiosis in Missouri Dogs: Eleven Cases. *J. Vet. Diagn. Invest.* **1994**, *6*, 380–382.
35. Miller, R. Gastrointestinal Phycomycosis in 63 Dogs. *J. Am. Vet. Med. Assoc.* **1985**, *186*, 473–478.
36. Kepler, D.; Cole, R.; Lee-Fowler, T.; Koehler, J.; Shrader, S.; Newton, J. Pulmonary Pythiosis in a Canine Patient. *Vet. Radiol. Ultrasound* **2019**, E20–E23.
37. Jaeger, G.H.; Rotstein, D.S.; Law, J.M. Prostatic Pythiosis in a Dog. *J. Vet. Intern. Med.* **2002**, *16*, 598–602.
38. Dowst, M.; Pavuk, A.; Vilela, R.; Vilela, C.; Mendoza, L. An Unusual Case of Cutaneous Feline Pythiosis. *Med. Mycol. Case Rep.* **2019**, *26*, 57–60.
39. Fortin, J.S.; Calcutt, M.J.; Kim, D.Y. Sublingual Pythiosis in a Cat. *Acta Vet. Scand.* **2017**, *59*, 63.
40. Duncan, D.; Hodgin, C.; Bauer, R.; Brignac, M. Cutaneous Pythiosis in Four Cats. In Proceedings of the Proceedings of the 43rd Meeting of the American College of Veterinary Pathologists; 1992; Vol. 29, p. 429.
41. Rakich, P.M.; Grooters, A.M.; Tang, K.N. Gastrointestinal Pythiosis in Two Cats. *J. Vet. Diagn. Invest.* **2005**, *17*, 262–269.
42. Bissonnette, K.; Sharp, N.; Dykstra, M.; Robertson, I.; Davis, B.; Padhye, A.; Kaufman, L. Nasal and Retrobulbar Mass in a Cat Caused by Pythium Insidiosum. *J. Med. Vet. Mycol.* **1991**, *29*, 39–44.
43. Grant, D.; Glass, R.; Hansen, R.; Vilela, R.; Mendoza, L. Cutaneous Pythiosis in a Red Brangus Beef Calf Cured by Immunotherapy. *Med. Mycol. Case Rep.* **2016**, *14*, 1–3.
44. Miller, R.; Olcott, B.; Archer, M. Cutaneous Pythiosis in Beef Calves. *J. Am. Vet. Med. Assoc.* **1985**, *186*, 984–986.
45. White, S.D.; Ghoddusi, M.; Grooters, A.M.; Jones, K. Cutaneous Pythiosis in a Nontravelled California Horse. *Vet. Dermatol.* **2008**, *19*, 391–394.
46. Sedrish, S.; Moore, R.; Valdes-Vasquez, M.; Haynes, P.; Vicek, T. Adjunctive Use of a Neodymium: Yttrium-Aluminum Garnet Laser for Treatment of Pythiosis Granulomas in Two Horses. *J. Am. Vet. Med. Assoc.* **1997**, *211*, 464–465.
47. Chaffin, M.; Schumacher, J.; Hooper, N. Multicentric Cutaneous Pythiosis in a Foal. *J. Am. Vet. Med. Assoc.* **1992**, *201*, 310–312.
48. Miller, R.I.; Wold, D.; Lindsay, W.A.; Beadle, R.E.; McClure, J.J.; McClure, J.R.; McCoy, D.J. Complications Associated with Immunotherapy of Equine Phycomycosis. *J. Am. Vet. Med. Assoc.* **1983**, *182*, 1227–1229.
49. Purcell, K.; Johnson, P.; Kreeger, J.; Wilson, D. Jejunal Obstruction Caused by a Pythium Insidiosum Granuloma in a Mare. *J. Am. Vet. Med. Assoc.* **1994**, *205*, 337–339.
50. Morton, L.; Morton, D.; Baker, G.; Gelberg, H. Chronic Eosinophilic Enteritis Attributed to Pythium Sp. in a Horse. *Vet. Pathol.* **1991**, *28*, 542–544.
51. Allison, N.; Gillis, J.; others Enteric Pythiosis in a Horse. *J. Am. Vet. Med. Assoc.* **1990**, *196*, 462–464.
52. Brown, C.C.; Roberts, E.D. Intestinal Pythiosis in a Horse. *Aust. Vet. J.* **1988**, *65*, 88–89.
53. Goad, M.P. Pulmonary Pythiosis in a Horse. *Vet. Pathol.* **1984**, *21*, 261–262.
54. Worster, A.A.; Lillich, J.D.; Cox, J.H.; Rush, B.R. Pythiosis with Bone Lesions in a Pregnant Mare. *J. Am. Vet. Med. Assoc.* **2000**, *216*, 1795–1798.

55. Eaton, S. Osseous Involvement by Pythium Insidiosum. *Compend. Contin. Educ. Pract. Vet.* **1993**, *15*, 485–488.
56. Nguyen, D.; Vilela, R.; Miraglia, B.M.; Vilela, G.; Jasem-Alali, N.; Rohn, R.; Glass, R.; Hansen, R.D.; Mendoza, L. Geographic Distribution of Pythium Insidiosum Infections in the United States. *J. Am. Vet. Med. Assoc.* **2021**. in press.
57. Foil, C.; Short, B.; Fadok, V.; Kunkle, G. A Report of Subcutaneous Pythiosis in Five Dogs and a Review of the Etiologic Agent Pythium Spp. *J. Am. Anim. Hosp. Assoc. USA* **1984**, *20*, 959–966.
58. Cooper Jr, R.; Allison, N.; Boring, J. Apparent Successful Surgical Treatment of Intestinal Pythiosis with Vascular Invasion in a Dog. *Canine Pract. USA* **1991**, *16*, 9–12.
59. Videla, R.; van Amstel, S.; O'Neill, S.H.; Frank, L.A.; Newman, S.J.; Vilela, R.; Mendoza, L. Vulvar Pythiosis in Two Captive Camels (Camelus Dromedarius). *Sabouraudia* **2012**, *50*, 212–224.
60. Wellehan, J.F.; Farina, L.L.; Keoughan, C.G.; Lafortune, M.; Grooters, A.M.; Mendoza, L.; Brown, M.; Terrell, S.P.; Jacobson, E.R.; Heard, D.J. Pythiosis in a Dromedary Camel (Camelus Dromedarius). *J. Zoo Wildl. Med.* **2004**, *35*, 564–568.
61. Pesavento, P.; Barr, B.; Riggs, S.; Eigenheer, A.; Pamma, R.; Walker, R. Cutaneous Pythiosis in a Nestling White-Faced Ibis. *Vet. Pathol.* **2008**, *45*, 538–541.
62. Buergelt, C.; Powe, J.; White, T. Abdominal Pythiosis in a Bengal Tiger (Panthera Tigris Tigris). *J. Zoo Wildl. Med.* **2006**, *37*, 186–189.
63. Camus, A.C.; Grooters, A.M.; Aquilar, R.F. Granulomatous Pneumonia Caused by Pythium Insidiosum in a Central American Jaguar, Panthera Onca. *J. Vet. Diagn. Invest.* **2004**, *16*, 567–571.
64. Jara, M.; Holcomb, K.; Wang, X.; Goss, E.M.; Machado, G. The Potential Distribution of Pythium Insidiosum in the Chincoteague National Wildlife Refuge, Virginia. *Front. Vet. Sci.* **2021**, *8*, 640339.
65. Schurko, A.; Mendoza, L.; de Cock, A.W.; Klassen, G.R. Evidence for Geographic Clusters: Molecular Genetic Differences among Strains of Pythium Insidiosum from Asia, Australia and the Americas Are Explored. *Mycologia* **2003**, *95*, 200–208.
66. de Moraes Gimenes Bosco, S.; Bagagli, E.; Araújo Jr, J.P.; Candeias, J.M.G.; De Franco, M.F.; Marques, M.E.A.; Mendoza, L.; de Camargo, R.P.; Marques, S.A. Human Pythiosis, Brazil. *Emerg. Infect. Dis.* **2005**, *11*, 715.
67. Lemos, G.; Petrucci, L.; Vieira, V.; di Filippo, P. Treatment of Equine Cutaneous Pythiosis with Triamcinolone Acetate and Potassium Iodide. *Rev. Acadêmica Ciênc. Anim.* **2018**, *16*, e162507.
68. Souto, E.P.F.; Maia, L.A.; Neto, E.G.M.; Kommers, G.D.; Junior, F.G.; Riet-Correa, F.; Galiza, G.J.; Dantas, A.F. Pythiosis in Equidae in Northeastern Brazil: 1985–2020. *J. Equine Vet. Sci.* **2021**, *105*, 103726.
69. da Silva, W.P.; Costa, R.V.C.; de Oliveira Henriques, M. Pitiose Cutânea Em Equino: Relato de Caso. *Rev. Saber Digit.* **2018**, *10*, 57–67.
70. Assis-Brasil, N.D. de; Marcolongo-Pereira, C.; Stigger, A.L.; Fiss, L.; Santos, B.L.; Coelho, A.C.B.; Sallis, E.S.V.; Fernandes, C.G.; Schild, A.L. Equine Dermatopathies in Southern Brazil: A Study of 710 Cases. *Ciênc. Rural* **2015**, *45*, 519–524.
71. Frey, F.; Velho, J.R.; Lins, L.A.; Nogueira, C.E.W.; Santurio, J.M. Pitiose Equina Na Região Sul Do Brasil. *Rev. Port. Cienc. Vet.* **2007**, *102*, 107–111.
72. Dória, R.G.; Carvalho, M.B.; Freitas, S.H.; Laskoski, L.M.; Colodel, E.M.; Mendonça, F.S.; Silva, M.A.; Grigoletto, R.; Neto, P.F. Evaluation of Intravenous Regional Perfusion with Amphotericin B and Dimethylsulfoxide to Treat Horses for Pythiosis of a Limb. *BMC Vet. Res.* **2015**, *11*, 152.
73. Galiza, G.J.; Silva, T.M. da; Caprioli, R.A.; Barros, C.S.; Irigoyen, L.F.; Fighera, R.A.; Lovato, M.; Kommers, G.D. Occurrence of Mycoses and Pythiosis in Domestic Animals: 230 Cases. *Pesqui. Veterinária Bras.* **2014**, *34*, 224–232.
74. Martins, T.; Kommers, G.; Trost, M.; Inkelmann, M.; Fighera, R.; Schild, A. A Comparative Study of the Histopathology and Immunohistochemistry of Pythiosis in Horses, Dogs and Cattle. *J. Comp. Pathol.* **2012**, *146*, 122–131.
75. dos Santos, C.E.; Ubiali, D.G.; Pescador, C.A.; Zanette, R.A.; Santurio, J.M.; Marques, L.C. Epidemiological Survey of Equine Pythiosis in the Brazilian Pantanal and Nearby Areas: Results of 76 Cases. *J. Equine Vet. Sci.* **2014**, *34*, 270–274.

76. dos Santos, C.E.P.; Santurio, J.M.; Marques, L.C. Pitiose Em Animais de Produção No Pantanal Matogrossense. *Pesqui. Veterinária Bras.* **2011**, *31*, 1083–1089.
77. Santos, C.E.P.; Santurio, J.M.; Colodel, E.M.; Juliano, R.S.; Silva, J.A.; Marques, L.C. Contribution to the Study of Cutaneous Pythiosis in Equidae from Northern Pantanal, Brazil. *Ars Vet.* **2011**, *27*, 134–140.
78. Doria, R.G.; Freitas, S.H.; Linardi, R.L.; de Souza Mendonça, F.; Arruda, L.P.; Boabaid, F.M.; Valadão, C.A. Treatment of Pythiosis in Equine Limbs Using Intravenous Regional Perfusion of Amphotericin B. *Vet. Surg.* **2012**, *41*, 759–765.
79. D'Avila, P.P.; Pacheco, C.D.V.; Pereira, R.C.; Daltro, D.S.; Ribas, L.M. Pitiose Equina Na Regiao Da Campanha Do Rio Grande Do Sul - Relato de Caso. *Rev. Cient. Eletronica Med. Vet.* **2011**, *9*, 1–8.
80. Prado, L.G.; Guedes, A.R.M.; Werneck, J.G.; Gomes, C.A.R.; Santos, F.A.; Anacleto, T.P.; Akamatsu, A.; Siqueira, L.J.R.; Malagó, R. Pitiose Cutânea Com Invasão de Articulação Em Equino. *R Bras Med Equina* **2015**, *10*, 10–13.
81. Salomão-Nascimento, R.B.; Frazão-Teixeira, E.; de Oliveira, F. Hepatic and Renal Analysis in Horses with Pythiosis Treated with Potassium Iodate, through the Detection of Serum Proteins, Nitrogenated Substances and Enzymes. *Rev. Bras. Med. Veterinária* **2010**, *32*, 105–110.
82. Bandeira, A.; Santos, J.; Melo, M.; Andrade, V.; Dantas, A.; Araujo, J. Pitiose Equina No Estado de Sergipe, Brasil. *Ciênc Vet Tróp Recife-PE* **2009**, *12*, 46–54.
83. de Faria Maciel, I.C.; Silveira, J.T.; Maia, C.A.; Sousa, R.M.; Oliveira, N.J.F.; Duarte, E.R. Pitiose Fatal Em Eqüino Tratado Inicialmente Para Habronemose Cutânea. *Acta Sci. Vet.* **2008**, *36*, 293–297.
84. De Moraes Gimenes Bosco, S.; Martins Reis, G.; Cordeiro Theodoro, R.; Assis Da Graça Macoris, S.; Alencar Marques, S.; Da Graça Macoris, D.; Bagagli, E. Morphological and Molecular Characterization of an Equine Isolate of Pythium Insidiosum and Comparison with the First Human Isolate from the Same Geographic Region. *Sabouraudia* **2008**, *46*, 557–565.
85. Pedroso, P.M.O.; Júnior, P.S.B.; Pescador, C.A.; Dalto, A.G.C.; da Costa, G.R.; Pereira, D.I.B.; Santurio, J.M.; Driemeier, D. Diagnóstico Imuno-Histoquímico de Pitiose Cutânea Em Equinos. *Acta Sci. Vet.* **2009**, *37*, 49–52.
86. Headley, S.A.; Arruda Junior, H.N. Equine Cutaneous Pythiosis: A Report of Four Cases. *Ciênc. Rural* **2004**, *34*, 289–292.
87. Sallis, E.S.V.; Pereira, D.I.B.; Raffi, M.B. Pitiose Cutânea Em Eqüinos: 14 Casos. *Ciênc. Rural* **2003**, *33*, 899–903.
88. Santurio, J.M.; Catto, J.B.; Leal, A.B.M.; Leal, A.T. *Tratamento Imunoterápico Da Pitiose Equina*; Embrapa Gado de Corte. Comunicado Técnico, 2001; Vol. 67;.
89. Rossato, C.; Fiss, L.; Sperotto, V.; Cardona, R.; Silva, R. Pythiosis with Atypical Location in the Soft Palate in a Horse in Southern Brazil. *Arq. Bras. Med. Veterinária E Zootec.* **2018**, *70*, 641–643.
90. da Paz, G.S.; Camargo, G.G.; Cury, J.E.; Apolonio, E.V.P.; Garces, H.G.; do Prado, A.C.; Chechi, J.L.; Oliveira, A.L.; Watanabe, M.J.; Bagagli, E.; et al. Outbreak of Equine Pythiosis in a Southeastern Region of Brazil: Environmental Isolation and Phylogeny. *Transbound. Emerg. Dis.* **2021**. in press.
91. Bezerra Júnior, P.S.; Pedroso, P.M.O.; Pavarini, S.P.; Dalto, A.G.C.; Santúrio, J.M.; Driemeier, D. Equine Intestinal Pythiosis in Southern Brazil. *Arq. Bras. Med. Veterinária E Zootec.* **2010**, *62*, 481–483.
92. Reis Jr, J.L.; de Carvalho, E.C.Q.; Nogueira, R.H.G.; Lemos, L.S.; Mendoza, L. Disseminated Pythiosis in Three Horses. *Vet. Microbiol.* **2003**, *96*, 289–295.
93. di Filippo, P.A.; Lemos, G.B.; Meireles, M.A.D.; Coutinho, Í.; Gobbi, F.P.; Godinho, A. Clinical and Anatomopathological Aspects of Bone Lesions Secondary to Pythium Insidiosum in Horses. *Rev. Acadêmica Ciênc. Anim.* **2020**, *18*, e18502.
94. Maia, L.A.; Souto, E.P.; Frade, M.T.; Pimentel, L.A.; Azevedo, E.O.; Kommers, G.D.; Riet-Correa, F.; Dantas, A.F. Pythiosis in Cattle in Northeastern Brazil. *Pesqui. Veterinária Bras.* **2020**, *40*, 340–345.
95. Konradt, G.; Bassuino, D.M.; Bianchi, M.V.; Castro, L.; Caprioli, R.A.; Pavarini, S.P.; Santurio, J.M.; Azevedo, M.I.; Jesus, F.P.; Driemeier, D. Cutaneous Pythiosis in Calves: An Epidemiologic, Pathologic, Serologic and Molecular Characterization. *Med. Mycol. Case Rep.* **2016**, *14*, 24–26.

96. Gabriel, A.L.; Kommers, G.D.; Trost, M.E.; Barros, C.S.; Pereira, D.B.; Schwendler, S.E.; Santurio, J.M. Outbreak of Cutaneous Pythiosis in Cattle. *Pesqui. Veterinária Bras.* **2008**, *28*, 583–587.
97. Grecco, F.B.; Schild, A.L.; Quevedo, P.; Assis-Brasil, N.D.; Kommers, G.D.; Marcolongo-Pereira, C.; Soares, M.P. Cutaneous Pythiosis in Cattle in the Southern Region of Rio Grande Do Sul, Brazil. *Pesqui. Veterinária Bras.* **2009**, *29*, 938–942.
98. Santurio, J.M.; Monteiro, A.B.; Leal, A.T.; Kommers, G.D.; de Sousa, R.S.; Catto, J.B. Cutaneous Pythiosis Insidiosi in Calves from the Pantanal Region of Brazil. *Mycopathologia* **1998**, *141*, 123–125.
99. Souto, E.; Maia, L.; Virgínio, J.; Carneiro, R.; Kommers, G.; Riet-Correa, F.; Galiza, G.; Dantas, A. Pythiosis in Cats in Northeastern Brazil. *J. Mycol. Médicale* **2020**, *30*, 101005.
100. Soares, L.M.C.; Schenkel, D.M.; Rosa, J.M.A.; Azevedo, L.S.; Tineli, T.R.; Dutra, V.; Colodel, E.M.; Pescador, C.A. Feline Subcutaneous Pythiosis. *Ciênc. Rural* **2019**, *49*, e20180448.
101. de Macêdo, L.B.; de Medeiros Oliveira, I.V.P.; Pimentel, M.M.L.; da Costa Reis, P.F.C.; de Macedo, M.F.; Filgueira, K.D. Primary Description of Pythiosis in Autochthonous Canine from the City of Mossoró, Rio Grande Do Norte, Brazil. *Rev. Bras. Hig. E Sanidade Anim.* **2014**, *8*, 88–109.
102. Frade, M.T.; Diniz, P.V.; Olinda, R.G.; Maia, L.A.; de Galiza, G.J.; de Souza, A.P.; da Nóbrega Neto, P.I.; Dantas, A.F. Pythiosis in Dogs in the Semiarid Region of Northeast Brazil. *Pesqui. Veterinária Bras.* **2017**, *37*, 485–490.
103. Firmino, M.; Frade, M.; Alves, R.; Maia, L.; Olinda, R.; Ximenes, R.; Souza, A.; Dantas, A. Intestinal Intussusception Secondary to Enteritis Caused by Pythium Insidiosum in a Bitch: Case Report. *Arq. Bras. Med. Veterinária E Zootec.* **2017**, *69*, 623–626.
104. Fujimori, M.; Lopes, E.R.; Lima, S.R.; de Paula, D.A.J.; de Almeida, A.; Colodel, E.M.; Pescador, C.A.; Néspoli, P.E.B.; Nakazato, L.; Dutra, V.; et al. Pythium Insidiosum Colitis in a Dog: Treatment and Clinical Outcome. *Ciênc. Rural* **2016**, *46*, 526–529.
105. Pereira, D.I.; Botton, S.A.; Azevedo, M.I.; Motta, M.A.; Lobo, R.R.; Soares, M.P.; Fonseca, A.O.; Jesus, F.P.; Alves, S.H.; Santurio, J.M. Canine Gastrointestinal Pythiosis Treatment by Combined Antifungal and Immunotherapy and Review of Published Studies. *Mycopathologia* **2013**, *176*, 309–315.
106. Fernandes, C.P.; Giordani, C.; Grecco, F.B.; Sallis, E.S.V.; Stainki, D.R.; Gaspar, L.F.J.; Ribeiro, C.L.G.; Nobre, M.O. Gastric Pythiosis in a Dog. *Rev. Iberoam. Micol.* **2012**, *29*, 235–237.
107. Stragliotto, A.; Pires, M.A.M.; Presser, C.I.; Ubiali, D.G.; Colodel, E.M.; Pescador, C.A. Granulomatous Enteritis by Pythium Insidiosum in Dog.; Florianópolis: Sociedade Brasileira de Medicina Veterinária, 2011; pp. 1–3.
108. da Nóbrega, D.F.; da Silva Junior, A.B.; Caleffo, T.; dos Santos Teruya, C.; da Cunha, O.; de Marco Viott, A. Enterite Piogranulomatosa Causada Por Pythium Insidiosum Em Cão. *Arch. Vet. Sci.* **2013**, *18*, 611–613.
109. Hunning, P.; Rigon, G.; Faraco, C.; Pavarini, S.; Sampaio, D.; Beheregaray, W.; Driemeier, D. Intestinal Obstruction by Pythium Insidiosum in a Dog: Case Report. *Arq. Bras. Med. Veterinária E Zootec.* **2010**, *62*, 801–805.
110. Souto, E.; Pessoa, C.; Pessoa, A.; Trost, M.; Kommers, G.; Correa, F.; Dantas, A. Esophageal Pythiosis in an Ostrich (Struthio Camelus). *Arq. Bras. Med. Veterinária E Zootec.* **2019**, *71*, 1081–1084.
111. Heck, L.C.; Bianchi, M.V.; Pereira, P.R.; Lorenzett, M.P.; de Lorenzo, C.; Pavarini, S.P.; Driemeier, D.; Sonne, L. Gastric Pythiosis in a Bactrian Camel (Bactrianus Camelus). *J. Zoo Wildl. Med.* **2018**, *49*, 784–787.
112. Maia, L.A.; Olinda, R.G.; Araújo, T.F.; Firmino, P.R.; Nakazato, L.; Neto, E.G.M.; Riet-Correa, F.; Dantas, A.F. Cutaneous Pythiosis in a Donkey (Equus Asinus) in Brazil. *J. Vet. Diagn. Invest.* **2016**, *28*, 436–439.
113. Do Carmo, P.; Portela, R.; Silva, T.; Oliveira-Filho, J.; Riet-Correa, F. Cutaneous Pythiosis in a Goat. *J. Comp. Pathol.* **2015**, *152*, 103–105.
114. Bernardo, F.D.; Conhizak, C.; Ambrosini, F.; de Jesus, F.P.; Santurio, J.M.; Kommers, G.D.; Elias, F.; Franciscato, C. Pythiosis in Sheep from Paraná, Southern Brazil. *Pesqui. Veterinária Bras.* **2015**, *35*, 513–517.

115. Ubiali, D.G.; Cruz, R.A.S.; De Paula, D.A.J.; Silva, M.C.; Mendonça, F.S.; Dutra, V.; Nakazato, L.; Colodel, E.M.; Pescador, C.A. Pathology of Nasal Infection Caused by Conidiobolus Lamprauges and Pythium Insidiosum in Sheep. *J. Comp. Pathol.* **2013**, *149*, 137–145.
116. Carrera, M.V.; Peixoto, R.M.; Gouveia, G.V.; Pessoa, C.R.; Jesus, F.P.; Santurio, J.M.; Botton, S.A.; Costa, M.M. Pitiose Em Ovinos Nos Estados de Pernambuco e Bahia. *Pesqui. Veterinária Bras.* **2013**, *33*, 476–482.
117. Portela, R. de A.; Riet-Correa, F.; Garino Junior, F.; Dantas, A.F.; Simões, S.V.; Silva, S. Diseases of the Nasal Cavity of Ruminants in Brazil. *Pesqui. Veterinária Bras.* **2010**, *30*, 844–854.
118. Santurio, J.; Argenta, J.; Schwendler, S.; Cavalheiro, A.; Pereira, D.; Zanette, R.; Alves, S.; Dutra, V.; Silva, M.; Arruda, L.; et al. Granulomatous Rhinitis Associated with Pythium Insidiosum Infection in Sheep. *Vet. Rec.* **2008**, *163*, 276–277.
119. Tabosa, I.; Riet-Correa, F.; Nobre, V.; Azevedo, E.; Reis-Junior, J.; Medeiros, R. Outbreaks of Pythiosis in Two Flocks of Sheep in Northeastern Brazil. *Vet. Pathol.* **2004**, *41*, 412–415.
120. Pessoa, C.R.; Riet-Correa, F.; Pimentel, L.A.; Garino Jr, F.; Dantas, A.F.; Kommers, G.D.; Tabosa, I.M.; Reis-Júnior, J.L. Pythiosis of the Digestive Tract in Sheep. *J. Vet. Diagn. Invest.* **2012**, *24*, 1133–1136.
121. Vilela, R.; Montalva, C.; Luz, C.; Humber, R.A.; Mendoza, L. Pythium Insidiosum Isolated from Infected Mosquito Larvae in Central Brazil. *Acta Trop.* **2018**, *185*, 344–348.
122. Bianchi, M.V.; Mello, L.S.; Lorenzo, C.D.; Lopes, B.C.; Snel, G.G.; Driemeier, D.; Pavarini, S.P. Lung Lesions of Slaughtered Horses in Southern Brazil. *Pesqui. Veterinária Bras.* **2018**, *38*, 2056–2064.
123. Guedes, R.; Zica, K.; Nogueira, R. Ficomicose e Habronemose Cutânea. Estudo Retrospectivo de Casos Diagnosticados No Período de 1979 a 1996. *Arq Bras Med Vet Zootec* **1998**, *50*, 465–468.
124. Neto, R.T.; De MG Bosco, S.; Amorim, R.L.; Brandão, C.V.; Fabris, V.E.; Estanislau, C.; Bagagli, E. Cutaneous Pythiosis in a Dog from Brazil. *Vet. Dermatol.* **2010**, *21*, 202–204.
125. Goloni, A.V.; Helayel, M.A.; Ramos, A.T.; Moroni, S.E.; Baptista, F.; Nunes, I.M.; et al Pitiose Rinofacial Em Ovino: Relato de Caso. *Arqs Pesq Anim* **2014**, *1*, 1–6.
126. Mustafa, V.S.; Guedes, K.M.; Lima, E.M.; Borges, J.R.; Castro, M.B. Doenças Da Cavidade Nasal Em Pequenos Ruminantes No Distrito Federal e No Estado de Goiás. *Pesqui. Veterinária Bras.* **2015**, *35*, 627–636.
127. Manço, M.H.; Ferreira, S.K.; Perossi, I.F.S.; Klein, M.; Pelógia, M.E.S.; Carra, G.J.U.; de Souza, C.D.D.; Costa, M.T.; Bosco, S.; Moraes, P.C.; et al. Metastatic Calcification and Granulomatous Grastroenteritis Associated to Pythium Insidiosum in a Dog. *Braz. J. Vet. Pathol.* **2021**, *14*, 50–55.
128. Viana, I.; Coutinho, I.; Gobbi, F.; Gaiotte, D.; Graca, F.; Costa, A.; et al Pitiose Equina: Descrição de 30 Casos Atendidos Na Universidade Estadual Do Norte Fluminense "Darcy Ribeiro", Campos Dos Goytacazes, RJ. In Proceedings of the Belo Horizonte: V e Z em Minas, Suplemento Especial; Belo Horizonte, MG, 25 abr 2015; Vol. 69–70.
129. Watanabe, M.J.; de Moura Alonso, J.; Alves, A.L.G.; Yamada, A.L.M.; de Moraes Gimenes Bosco, S.; Rodrigues, C.A.; Hussni, C.A. Equine Pythiosis: Report of 28 Cases from São Paulo State, Brazil. *Semina Ciênc. Agrár.* **2015**, *36*, 909–915.
130. d' Utra Vaz, B. Ptiose Nasal Em Equino. *Med. Veterinária* **2009**, *3*, 27–32.
131. Dias, D.; Doria, R.; Pereira, R.; Canola, P.; Di Filippo, P.; et al Topical Treatment Using Amphotericin B and DMSO for an Atypically Located Equine Cutaneous Pythiosis. *Acta Sci. Vet.* **2012**, *40*, 1088.
132. Torres, L.M.; Dantas, A.K.; Silva, J.K.; Araújo, K.N.; Junior, F.G.; Mendes, R.S. PITIOSE CUTÂNEA CANINA–RELATO DE CASO. *Ars Vet.* **2015**, *30*, 77–82.
133. dos Santos, G.F.; de Castro Borba Santos Junior, J. Pitiose Cutanea Em Equino: Relato de Caso. *Rev. Saber Digit.* **2020**, *12*, 149–158.
134. Tavares, T.R.; Frias, N.C.; Lopes, N.C.; Maldos, P.C.W.; Galvão-Dias, M.A.; Reis-Menezes, A.A. Equine Pitiosis in São Paulo: Case Report.; Pernambuco, Brazil, August 14 2013; p. 1.

135. de Almeida Sampaio, A.J.S.; Gomes, R.G.; Cosenza, M. Utilização de Imunoterápico No Tratamento Da Pitiose Equina. *Arq Ciênc Vet Zool UNIPAR* **2016**, *19*, 165–169.
136. Reis-Gomes, A.; Marcolongo-Pereira, C.; Sallis, E.S.V.; Bruhn, F.R.; Faria, R.O.; Schild, A.L.; Meireles, M.C. Epidemiology of Mycoses, Pitiosis and Micotoxicosis in Horses in Southeastern Rio Grande Do Sul, Brazil. *Pesqui. Veterinária Bras.* **2018**, *38*, 1110–1116.
137. Marques, S.A.; Bagagli, E.; Bosco, S.M.G.; Camargo, R.M.P.; Marques, M.E.A. Pythium Insidiosum: Report of the First Case of Human Infection in Brazil. *Bras Dermatol* **2006**, *81*, 483–5.
138. Souto, E.; Maia, L.; Olinda, R.; Galiza, G.; Kommers, G.; Miranda-Neto, E.; Dantas, A.; Riet-Correa, F. Pythiosis in the Nasal Cavity of Horses. *J. Comp. Pathol.* **2016**, *155*, 126–129.
139. de Souto, E.P.F.; Maia, L.Â.; Assis, D.M.; de Miranda Neto, E.G.; Kommers, G.D.; de Galiza, G.J.N.; Riet-Correa, F.; Dantas, A.F.M. Mastitis by Pythium Insidiosum in Mares. *Acta Sci. Vet.* **2019**, *47*, 387.
140. Pessoa, A.F.A.; Pessoa, C.R.M.; Miranda Neto, E.G.; Dantas, A.F.M.; Riet-Correa, F. Doenças de Pele Em Equídeos No Semiárido Brasileiro. *Pesqui. Veterinária Bras.* **2014**, *34*, 743–748.
141. Tabosa, I.; Medeiros, V.; Dantas, A.; Azevedo, E.; Maia, J. Pitiose Cutânea Em Eqüídeos No Semi-Árido Da Paraíba. *Arq Bras Med Vet Zootec* **1999**, *51*, 27–30.
142. Marcolongo-Pereira, C.; Sallis, E.S.V.; Raffi, M.B.; Pereira, D.I.B.; Hinnah, F.L.; Coelho, A.C.B.; Schild, A.L. Epidemiology of Equine Pythiosis in Southern of Rio Grande Do Sul State, Brazil. *Pesqui. Veterinária Bras.* **2012**, *32*, 865–868.
143. Meireles, M.C.A.; Riet-Correa, F.; Fischman, O.; Zambrano, A.F.H.; Zambrano, M.S.; Ribeiro, G.A. Cutaneous Pythiosis in Horses from Brazil. *Mycoses* **1993**, *36*, 139–142.
144. Souza, T.M.; Brum, J.S.; Fighera, R.A.; Brass, K.E.; Barros, C.S. Prevalence of Equine Skin Tumors Diagnosed at the Laboratory of Veterinary Pathology of the Federal University Santa Maria, Rio Grande Do Sul, Brazil. *Pesqui. Veterinária Bras.* **2011**, *31*, 379–382.
145. Pierezan, F.; Rissi, D.R.; Rech, R.R.; Fighera, R.A.; Brum, J.S.; Barros, C.S. Necropsy Findings Related to the Cause of Death in 335 Horses: 1968-2007. *Pesqui. Veterinária Bras.* **2009**, *29*, 275–280.
146. dos Santos, C.E.P.; Marques, L.C.; Zanette, R.A.; Jesus, F.P.K.; Santurio, J.M. Does Immunotherapy Protect Equines from the Reinfection by the Oomycete Pythium Insidiosum? *Clin. Vaccine Immunol.* **2011**, *18*, 1397–1399.
147. dos Santos, C.E.P.; Juliano, R.S.; Santurio, J.M.; Marques, L.C. Eficácia Da Imunoterapia No Tratamento de Pitiose Facial Em Equino. *Acta Sci. Vet.* **2011**, *39*, 955.
148. Leal, A.B.M.; Leal, A.T.; Santurio, J.M.; Kommers, G.D.; Catto, J.B. Pitiose Eqüina No Pantanal Brasileiro: Aspectos Clínico-Patológicos de Casos Típicos e Atípicos. *Pesqui. Veterinária Bras.* **2001**, *21*, 151–156.
149. Dalto, A.G.C.; Junior, P.S.B.; Miguel, P. Pitiose Intestinal Em Um Equino No Rio Grande Do Sul. *Rev Univ Rural* **2007**, *27*, 131–133.
150. Trost, M.E.; Gabriel, A.L.; Masuda, E.K.; Fighera, R.A.; Irigoyen, L.F.; Kommers, G.D.; others Clinical, Morphologic and Immunohistochemical Aspects of Canine Gastrintestinal Pythiosis. *Pesqui. Veterinária Bras.* **2009**, *29*, 673–679.
151. Rodrigues, A.; Graça, D.; Fontoura, C.; Cavalheiro, A.; Henzel, A.; Schwendler, S.; Alves, S.; Santurio, J. Intestinal Dog Pythiosis in Brazil. *J. Mycol. Médicale* **2006**, *16*, 37–41.
152. Maroneze, B.P.; Botton, S.A.; Mota, M.A.; Lobo, R.R.; Soares, M.P.; Valente, J.S.S.; Azevedo, M.I.; Ribeiro, T.C.; Sallis, E.S.V.; da Silveira, D.H.; et al. Terapia Combinada No Tratamento de Pitiose Gastrointestinal Em Um Canino. *Acta Sci Vet* **2012**, *40*, s36.
153. Rech, R.; Graça, D.; Barros, C. Pitiose Em Um Cão: Relato de Caso e Diagnósticos Diferenciais. *Clínica Veterinária* **2004**, *50*, 68–72.

154. Pereira, D.I.B.; Schild, A.L.; Motta, M.A.; Fighera, R.A.; Sallis, E.S.V.; Marcolongo-Pereira, C. Cutaneous and Gastrointestinal Pythiosis in a Dog in Brazil. *Vet. Res. Commun.* **2010**, *34*, 301–306.
155. Ubiali, D.G.; Pereira, A.H.; Boabaid, F.M.; Dutra, V.; Nakazato, L.; Campos, C.G.; Colodel, E.M.; Pescador, C.A.; Riet-Correa, F. Successful Potassium Iodide Treatment for Rhinofacial Pythiosis in Sheep. *J. Med. Mycol.* **2022**, *32*, 101233.
156. Hasika, R.; Lalitha, P.; Radhakrishnan, N.; Rameshkumar, G.; Prajna, N.V.; Srinivasan, M. Pythium Keratitis in South India: Incidence, Clinical Profile, Management, and Treatment Recommendation. *Indian J. Ophthalmol.* **2019**, *67*, 42–47.
157. Appavu, S.P.; Prajna, L.; Rajapandian, S.G.K. Genotyping and Phylogenetic Analysis of Pythium Insidiosum Causing Human Corneal Ulcer. *Med. Mycol.* **2020**, *58*, 211–218.
158. Chatterjee, S.; Agrawal, D. Azithromycin in the Management of Pythium Insidiosum Keratitis. *Cornea* **2018**, *37*, e8–e9.
159. Agarwal, S.; Iyer, G.; Srinivasan, B.; Benurwar, S.; Agarwal, M.; Narayanan, N.; Lakshmipathy, M.; Radhika, N.; Rajagopal, R.; Krishnakumar, S.; et al. Clinical Profile, Risk Factors and Outcome of Medical, Surgical and Adjunct Interventions in Patients with Pythium Insidiosum Keratitis. *Br. J. Ophthalmol.* **2019**, *103*, 296–300.
160. Bagga, B.; Sharma, S.; Guda, S.J.M.; Nagpal, R.; Joseph, J.; Manjulatha, K.; Mohamed, A.; Garg, P. Leap Forward in the Treatment of Pythium Insidiosum Keratitis. *Br. J. Ophthalmol.* **2018**, *102*, 1629–1633.
161. Sharma, S.; Balne, P.K.; Motukupally, S.R.; Das, S.; Garg, P.; Sahu, S.K.; Arunasri, K.; Manjulatha, K.; Mishra, D.K.; Shivaji, S. Pythium Insidiosum Keratitis: Clinical Profile and Role of DNA Sequencing and Zoospore Formation in Diagnosis. *Cornea* **2015**, *34*, 438–442.
162. Bagga, B.; Kate, A.; Mohamed, A.; Sharma, S.; Das, S.; Mitra, S. Successful Strategic Management of Pythium Insidiosum Keratitis with Antibiotics. *Ophthalmology* **2020**, *128*, 169–172.
163. Raghavan, A.; Bellamkonda, P.; Mendoza, L.; Rammohan, R. Pythium Insidiosum and Acanthamoeba Keratitis in a Contact Lens User. *BMJ Case Rep.* **2018**, *11*, e226386.
164. Gurnani, B.; Christy, J.; Narayana, S.; Rajkumar, P.; Kaur, K.; Gubert, J. Retrospective Multifactorial Analysis of Pythium Keratitis and Review of Literature. *Indian J. Ophthalmol.* **2021**, *69*, 1095–1101.
165. Mittal, R.; Jena, S.K.; Desai, A.; Agarwal, S. Pythium Insidiosum Keratitis: Histopathology and Rapid Novel Diagnostic Staining Technique. *Cornea* **2017**, *36*, 1124–1132.
166. Vishwakarma, P.; Mohanty, A.; Kaur, A.; Das, S.; Priyadarshini, S.R.; Mitra, S.; Mittal, R.; Sahu, S.K. Pythium Keratitis: Clinical Profile, Laboratory Diagnosis, Treatment, and Histopathology Features Post-treatment at a Tertiary Eye Care Center in Eastern India. *Indian J Ophthalmol* **2021**, *69*, 1544–1552.
167. Rathi, A.; Chakrabarti, A.; Agarwal, T.; Pushker, N.; Patil, M.; Kamble, H.; Titiyal, J.S.; Mohan, R.; Kashyap, S.; Sharma, S.; et al. Pythium Keratitis Leading to Fatal Cavernous Sinus Thrombophlebitis. *Cornea* **2018**, *37*, 519–522.
168. Kate, A.; Bagga, B.; Ahirwar, L.K.; Mishra, D.K.; Sharma, S. Unusual Presentation of Pythium Keratitis as Peripheral Ulcerative Keratitis: Clinical Dilemma. *Ocul. Immunol. Inflamm.* **2021**. in press.
169. Agarwal, S.; Srinivasan, B.; Janakiraman, N.; Therese, L.K.; KrishnaKumar, S.; Patel, N.; Thenmozhi, V.; Iyer, G.; et al Role of Topical Ethanol in the Treatment of Pythium Insidiosum Keratitis—a Proof of Concept. *Cornea* **2020**, *39*, 1102–1107.
170. Otta, S.K.; Praveena, P.E.; Raj, R.A.; Saravanan, P.; Priya, M.S.; Amarnath, C.B.; Bhuvaneswari, T.; Panigrahi, A.; Ravichandran, P. Pythium Insidiosum as a New Opportunistic Fungal Pathogen for Pacific White Shrimp, Litopenaeus Vannamei. *Indian J. Geo Mar. Sci.* **2018**, *47*, 1036–1041.
171. Agarwal, S.; Iyer, G.; Srinivasan, B.; Agarwal, M.; Kumar, S.P.S.; Therese, L.K. Clinical Profile of Pythium Keratitis: Perioperative Measures to Reduce Risk of Recurrence. *Br. J. Ophthalmol.* **2018**, *102*, 153–157.
172. Ramappa, M.; Nagpal, R.; Sharma, S.; Chaurasia, S. Successful Medical Management of Presumptive Pythium Insidiosum Keratitis. *Cornea* **2016**, *36*, 511–514.

173. Gurnani, B.; Narayana, S.; Christy, J.; Rajkumar, P.; Kaur, K.; Gubert, J. Successful Management of Pediatric Pythium Insidiosum Keratitis with Cyanoacrylate Glue, Linezolid, and Azithromycin: Rare Case Report. *Eur. J. Ophthalmol.* **2021**. in press.
174. Permpalung, N.; Worasilchai, N.; Manothummetha, K.; Torvorapanit, P.; Ratanawongphaibul, K.; Chuleerarux, N.; Plongla, R.; Chindamporn, A. Clinical Outcomes in Ocular Pythiosis Patients Treated with a Combination Therapy Protocol in Thailand: A Prospective Study. *Med. Mycol.* **2019**, *57*, 923–928.
175. Anutarapongpan, O.; Thanathanee, O.; Worrawitchawong, J.; Suwan-Apichon, O. Role of Confocal Microscopy in the Diagnosis of Pythium Insidiosum Keratitis. *Cornea* **2018**, *37*, 156–161.
176. Kosrirukvongs, P.; Chaiprasert, A.; Canyuk, C.; Wanachiwanawin, W. Comparison of Nested PCR and Culture Identification of Pythium Insidiosum in Patients with Pythium Keratitis. *J. Med. Assoc. Thail. Chotmaihet Thangphaet* **2016**, *99*, 1033–1038.
177. Lelievre, L.; Borderie, V.; Garcia-Hermoso, D.; Brignier, A.C.; Sterkers, M.; Chaumeil, C.; Lortholary, O.; Lanternier, F. Imported Pythium Insidiosum Keratitis after a Swim in Thailand by a Contact Lens-Wearing Traveler. *Am. J. Trop. Med. Hyg.* **2015**, *92*, 270–273.
178. Thanathanee, O.; Enkvetchakul, O.; Rangsin, R.; Waraasawapati, S.; Samerpitak, K.; Suwan-apichon, O. Outbreak of Pythium Keratitis during Rainy Season: A Case Series. *Cornea* **2013**, *32*, 199–204.
179. Lekhanont, K.; Chuckpaiwong, V.; Chongtrakool, P.; Aroonroch, R.; Vongthongsri, A. Pythium Insidiosum Keratitis in Contact Lens Wear: A Case Report. *Cornea* **2009**, *28*, 1173–1177.
180. Krajaejun, T.; Sathapatayavongs, B.; Pracharktam, R.; Nitiyanant, P.; Leelachaikul, P.; Wanachiwanawin, W.; Chaiprasert, A.; Assanasen, P.; Saipetch, M.; Mootsikapun, P.; et al. Clinical and Epidemiological Analyses of Human Pythiosis in Thailand. *Clin. Infect. Dis.* **2006**, *43*, 569–576.
181. Narkwiboonwong, T.; Watanakijthavonkul, K.; Paocharern, P.; Singsakul, A.; Wongsa, A.; Woracharoensri, N.; Worasilchai, N.; Chindamporn, A.; Panoi, A.; Methipisit, T.; et al. Cerebral Pythiosis: A Case Report of Pythium Insidiosum Infection Presented with Brain Abscess. *J Infect Antimicrob Agents* **2011**, *28*, 129–132.
182. Nonpassopon, M.; Jongkhajornpong, P.; Aroonroch, R.; Koovisitsopit, A.; Lekhanont, K. Predisposing Factors, Clinical Presentations, and Outcomes of Contact Lens-Related Pythium Keratitis. *Cornea* **2021**, *40*, 1413–1419.
183. Dhirachaikulpanich, D.; Soraprajum, K.; Boonsopon, S.; Pinitpuwadol, W.; Lourthai, P.; Punyayingyong, N.; Tesavibul, N.; Choopong, P. Epidemiology of Keratitis/Scleritis-Related Endophthalmitis in a University Hospital in Thailand. *Sci. Rep.* **2021**, *11*, 11217.
184. Susaengrat, N.; Torvorapanit, P.; Plongla, R.; Chuleerarux, N.; Manothummetha, K.; Tuangsirisup, J.; Worasilchai, N.; Chindamporn, A.; Permpalung, N. Adjunctive Antibacterial Agents as a Salvage Therapy in Relapsed Vascular Pythiosis Patients. *Int. J. Infect. Dis.* **2019**, *88*, 27–30.
185. Chitasombat, M.N.; Larbcharoensub, N.; Chindamporn, A.; Krajaejun, T. Clinicopathological Features and Outcomes of Pythiosis. *Int. J. Infect. Dis.* **2018**, *71*, 33–41.
186. Worasilchai, N.; Permpalung, N.; Chongsathidkiet, P.; Leelahavanichkul, A.; Mendoza, A.L.; Palaga, T.; Reantragoon, R.; Finkelman, M.; Sutcharitchan, P.; Chindamporn, A. Monitoring Anti-Pythium Insidiosum IgG Antibodies and (1→3)-β-D-Glucan in Vascular Pythiosis. *J. Clin. Microbiol.* **2018**, *56*, e00610-18.
187. Sermsathanasawadi, N.; Praditsuktavorn, B.; Hongku, K.; Wongwanit, C.; Chinsakchai, K.; Ruangsetakit, C.; Hahtapornsawan, S.; Mutirangura, P. Outcomes and Factors Influencing Prognosis in Patients with Vascular Pythiosis. *J. Vasc. Surg.* **2016**, *64*, 411–417.
188. Reanpang, T.; Orrapin, S.; Orrapin, S.; Arworn, S.; Kattipatanapong, T.; Srisuwan, T.; Vanittanakom, N.; Lekawanvijit, S.P.; Rerkasem, K. Vascular Pythiosis of the Lower Extremity in Northern Thailand: Ten Years' Experience. *Int. J. Low. Extrem. Wounds* **2015**, *14*, 245–250.

189. Keoprasom, N.; Chularojanamontri, L.; Chayakulkeeree, M.; Chaiprasert, A.; Wanachiwanawin, W.; Ruangsetakit, C. Vascular Pythiosis in a Thalassemic Patient Presenting as Bilateral Leg Ulcers. *Med. Mycol. Case Rep.* **2013**, *2*, 25–28.
190. Laohapensang, K.; Rutherford, R.B.; Supabandhu, J.; Vanittanakom, N. Vascular Pythiosis in a Thalassemic Patient. *Vascular* **2009**, *17*, 234–238.
191. Torvorapanit, P.; Chuleerarux, N.; Plongla, R.; Worasilchai, N.; Manothummetha, K.; Thongkam, A.; Langsiri, N.; Diewsurin, J.; Kongsakpaisan, P.; Bansong, R.; et al. Clinical Outcomes of Radical Surgery and Antimicrobial Agents in Vascular Pythiosis: A Multicenter Prospective Study. *J. Fungi* **2021**, *7*, 114.
192. Pathomsakulwong, W.; Laikul, A.; Theerawatanasirikul, S.; Limsivilai, O.; Yurayart, C.; Phetudomsinsuk, K. Equine Pythiosis in Thailand. In Proceedings of the 57th Kasetsart University Annual Conference, Bangkok (Thailand); February 29 2019; pp. 1–6.
193. Tonpitak, W.; Pathomsakulwong, W.; Sornklien, C.; Krajaejun, T.; Wutthiwithayaphong, S. First Confirmed Case of Nasal Pythiosis in a Horse in Thailand. *JMM Case Rep.* **2018**, *5*, e005136.
194. Chindamporn, A.; Kammarnjessadakul, P.; Kesdangsakonwut, S.; Banlunara, W. A Case of Canine Cutaneous Pythiosis in Thailand. *Access Microbiol.* **2020**, *2*, acmi000109.
195. Rotchanapreeda, T.; Sae-Chew, P.; Lohnoo, T.; Yingyong, W.; Rujirawat, T.; Kumsang, Y.; Payattikul, P.; Jaturapaktrarak, C.; Intaramat, A.; Pathomsakulwong, W.; et al. Immunological Cross-Reactivity of Proteins Extracted from the Oomycete Pythium Insidiosum and the Fungus Basidiobolus Ranarum Compromises the Detection Specificity of Immunodiagnostic Assays for Pythiosis. *J. Fungi* **2021**, *7*, 474.
196. Permpalung, N.; Worasilchai, N.; Plongla, R.; Upala, S.; Sanguankeo, A.; Paitoonpong, L.; Mendoza, L.; Chindamporn, A. Treatment Outcomes of Surgery, Antifungal Therapy and Immunotherapy in Ocular and Vascular Human Pythiosis: A Retrospective Study of 18 Patients. *J. Antimicrob. Chemother.* **2015**, *70*, 1885–1892.
197. Puangsricharern, V.; Chotikkakamthorn, P.; Tulvatana, W.; Kittipibul, T.; Chantaren, P.; Reinprayoon, U.; Kasetsuwan, N.; Satitpitakul, V.; Worasilchai, N.; Chindamporn, A. Clinical Characteristics, Histopathology, and Treatment Outcomes of Pythium Keratitis: A Retrospective Cohort Study. *Clin. Ophthalmol. Auckl. NZ* **2021**, *15*, 1691–1701.
198. Thianprasit, M.; Chaiprasert, A.; Imwidthaya, P. Human Pythiosis. *Curr. Top. Med. Mycol.* **1996**, *7*, 43–54.
199. Krajaejun, T.; Pracharktam, R.; Wongwaisayawan, S.; Rochanawutinon, M.; Kunakorn, M.; Kunavisarut, S. Ocular Pythiosis: Is It under-Diagnosed? *Am. J. Ophthalmol.* **2004**, *137*, 370–372.
200. Kunavisarut, S.; Nimvorapan, T.; Methasiri, S. Pythium Corneal Ulcer in Ramathibodi Hospital. *J. Med. Assoc. Thai.* **2003**, *86*, 338–342.
201. Tanphaichitra, D. Tropical Disease in the Immunocompromised Host: Melioidosis and Pythiosis. *Rev. Infect. Dis.* **1989**, *11*, S1629–S1643.
202. Imwidthaya, P. Human Pythiosis in Thailand. *Postgrad. Med. J.* **1994**, *70*, 558–560.
203. Khunkhet, S.; Rattanakaemakorn, P.; Rajatanavin, N. Pythiosis Presenting with Digital Gangrene and Subcutaneous Nodules Mimicking Medium Vessel Vasculitis. *JAAD Case Rep.* **2015**, *1*, 399–402.
204. Hahtapornsawan, S.; Wongwanit, C.; Chinsakchai, K.; Hongku, K.; Sermsathanasawadi, N.; Ruangsetakit, C.; Mutirangura, P. Suprainguinal Vascular Pythiosis: Effective Long-Term Outcome of Aggressive Surgical Eradication. *Ann. Vasc. Surg.* **2014**, *28*, 1797.e1-6.
205. Sudjaritruk, T.; Sirisanthana, V. Successful Treatment of a Child with Vascular Pythiosis. *BMC Infect. Dis.* **2011**, *11*, 33.
206. Sathapatayavongs, B.; Leelachaikul, P.; Prachaktam, R.; Atichartakarn, V.; Sriphojanart, S.; Trairatvorakul, P.; Jirasiritham, S.; Nontasut, S.; Eurvilaichit, C.; Flegel, T. Human Pythiosis Associated with Thalassemia Hemoglobinopathy Syndrome. *J. Infect. Dis.* **1989**, *159*, 274–280.

207. Chetchotisakd, P.; Pairojkul, C.; Porntaveevudhi, O.; Sathapatayavongs, B.; Mairiang, P.; Nuntirooj, K.; Patjanasoontorn, B.; Saew, O.; Chaiprasert, A.; Haswell-Elkins, M. Human Pythiosis in Srinagarind Hospital: One Year's Experience. *J. Med. Assoc. Thai.* **1992**, *75*, 248–254.
208. Wanachiwanawin, W.; Thianprasit, M.; Fucharoen, S.; Chaiprasert, A.; Na Ayudhya, N.S.; Sirithanaratkul, N.; Piankijagum, A. Fatal Arteritis Due to Pythium Insidiosum Infection in Patients with Thalassaemia. *Trans. R. Soc. Trop. Med. Hyg.* **1993**, *87*, 296–298.
209. Thitithanyanont, A.; Mendoza, L.; Chuansumrit, A.; Pracharktam, R.; Laothamatas, J.; Sathapatayavongs, B.; Lolekha, S.; Ajello, L. Use of an Immunotherapeutic Vaccine to Treat a Life-Threatening Human Arteritic Infection Caused by Pythium Insidiosum. *Clin. Infect. Dis.* **1998**, *27*, 1394–1400.
210. Prasertwitayakij, N.; Louthrenoo, W.; Kasitanon, N.; Thamprasert, K.; Vanittanakom, N. Human Pythiosis, a Rare Cause of Arteritis: Case Report and Literature Review. *Semin Arthritis Rheum* **2003**, *33*, 204–214.
211. Laohapensang, K.; Rerkasem, K.; Supabandhu, J.; Vanittanakom, N. Necrotizing Arteritis Due to Emerging Pythium Insidiosum Infection in Patients with Thalassemia: Rapid Diagnosis with PCR and Serological Tests. *Int. J. Angiol.* **2005**, *14*, 123–128.
212. Wanachiwanawin, W.; Mendoza, L.; Visuthisakchai, S.; Mutsikapan, P.; Sathapatayavongs, B.; Chaiprasert, A.; Suwanagool, P.; Manuskiatti, W.; Ruangsetakit, C.; Ajello, L. Efficacy of Immunotherapy Using Antigens of Pythium Insidiosum in the Treatment of Vascular Pythiosis in Humans. *Vaccine* **2004**, *22*, 3613–3621.
213. Pupaibool, J.; Chindamporn, A.; Patarakul, K.; Suankratay, C.; Sindhuphak, W.; Kulwichit, W. Human Pythiosis. *Emerg. Infect. Dis.* **2006**, *12*, 517.
214. Chitasombat, M.N.; Petchkum, P.; Horsirimanont, S.; Sornmayura, P.; Chindamporn, A.; Krajaejun, T. Vascular Pythiosis of Carotid Artery with Meningitis and Cerebral Septic Emboli: A Case Report and Literature Review. *Med. Mycol. Case Rep.* **2018**, *21*, 57–62.
215. Tantisarasart, T.; Patchimkul, P.; Wongbunterng, S.; Rangsipanuratn, W. Keratic Pythiosis in Songklanagarind Hospital. *Thai J Ophthalmol* **1993**, *7*, 39–45.
216. Badenoch, P.R.; Mills, R.A.; Chang, J.H.; Sadlon, T.A.; Klebe, S.; Coster, D.J. Pythium Insidiosum Keratitis in an Australian Child. *Clin. Experiment. Ophthalmol.* **2009**, *37*, 806–809.
217. Triscott, J.A.; Weedon, D.; Cabana, E. Human Subcutaneous Pythiosis. *J. Cutan. Pathol.* **1993**, *20*, 267–271.
218. Krockenberger, M.; Swinney, G.; Martin, P.; Rothwell, T.; Malik, R. Sequential Opportunistic Infections in Two German Shepherd Dogs. *Aust. Vet. J.* **2011**, *89*, 9–14.
219. Connole, M.D. Review of Animal Mycoses in Australia. *Mycopathologia* **1990**, *111*, 133–164.
220. Miller, R.I.; Campbell, R.S.F. Clinical Observations on Equine Phycomycosis. *Aust. Vet. J.* **1982**, *58*, 221–226.
221. Dowling, B.; Dart, A.; Kessell, A.; Pascoe, R.; Hodgson, D. Cutaneous Phycomycosis in Two Horses. *Aust. Vet. J.* **1999**, *77*, 780–783.
222. Miller, R. Treatment of Equine Phycomycosis by Immunotherapy and Surgery. *Aust. Vet. J.* **1981**, *57*, 377–382.
223. Cardona, J.; Vargas-Viloria, M.; Perdomo, S. Frequency of Cutaneous Pythiosis in Dairy Horses in Farms of Cordoba, Colombia. *Rev. Fac. Med. Vet. Zootec.* **2014**, *61*, 31–43.
224. Álvarez, J.C.; García, L.R.; Garay, O.V. Pythiosis Cutánea Equina En Córdoba, Colombia. Reporte de Cinco Casos. *Rev. Científica* **2010**, *20*, 590–594.
225. Álvarez, J.A.C.; Vergara, D.M.; Bossa, B.J. Pythiosis Mamaria En Una Yegua Criolla Colombiana. *Rev. Colomb. Cienc. Anim.* **2021**, *13*, e867.
226. Álvarez, J.A.C.; Viloria, M.V.; Ayola, S.C.P. Frequency of Presentation of Bovine Cutaneous Pythiosis (Pythium Insidiosum) in Three Cattle Farms in Cordoba, Colombia. *Rev CES Med. Vet. Zootec.* **2012**, *7*, 47–54.

227. Acero-Mondragón, E.J.; Rosas, D.A.B.; Arango, M.I.M. Mycoses for Pythium Insidiosum. First Case with Definitive Diagnosis in Colombia. *Nova Publ. Científica En Cienc. Bioméd.* **2013**, *11*, 65–72.
228. Baldrich Romero, N.E.; Patiño Quiroz, B.; Peña, H.; Juan Carlos, C.R. Primer Reporte de Pythiosis En Área Rual de Florencia-Caquetá. *Rev. Electron. Vet.* **2016**, *17*, 1–10.
229. Álvarez, J.A.C.; Socarrás, T.O.; Tóus, M.G. Dermopatías En Burros de Trabajo (Equus Asinus) En Áreas Rurales de Córdoba (Colombia). *Rev. Med. Vet.* **2017**, *34 supl*, 81–92.
230. Cardona-Álvarez, J.; Vargas-Vilória, M.; Patarroyo-Salcedo, J. Cutaneous Pythiosis in Horses Treated with Triamcinolone Acetonide. Part 1. Clinical Characterization. *Rev. MVZ Córdoba* **2016**, *21*, 5511–5524.
231. Álvarez, J.A.C.; Viloria, M.I.V.; Ayola, S.C.P. Clinical and Histopathological Evaluation of Cutaneous Pythiosis in Donkeys (Equus Asinus). *Rev. Med. Vet.* **2013**, *25*, 9–19.
232. Tartor, Y.H.; Hamad, M.H.; Abouzeid, N.Z.; El-Belkemy, F.A. Equine Pythiosis in Egypt: Clinicopathological Findings, Detection, Identification and Genotyping of Pythium Insidiosum. *Vet. Dermatol.* **2020**, *31*, 298-e73.
233. Elkhenany, H.; Nabil, S.; Abu-Ahmed, H.; Mahmoud, H.; Korritum, A.; Khalifa, H. Treatment and Outcome of Horses with Cutaneous Pythiosis, and Meta-Analysis of Similar Reports. *Slov. Vet. Res.* **2019**, *56*, 281–291.
234. Awadin, W.; Mosbah, E.; Zaghloul, A.; Loreto, É.; Zanette, R. Scrotal Pythiosis in a Draft Horse. *J Vet Sci Med Diagn* **2013**, *2*, 1000120.
235. Mosbah, E.; Karrouf, G.I.; Younis, E.A.; Saad, H.S.; Ahdy, A.; Zaghloul, A.E. Diagnosis and Surgical Management of Pythiosis in Draft Horses: Report of 33 Cases in Egypt. *J. Equine Vet. Sci.* **2012**, *32*, 164–169.
236. Atiba, A.; Ghazy, A.; Hamad, M. Evaluating the Efficacy of Surgical Excision and Topical Dimethyl Sulphoxide (DMSO) in the Treatment of Equine Cutaneous Pythiosis. *Iran. J. Vet. Res.* **2020**, *21*, 301–307.
237. Salas, Y.; Márquez, A.; Canelón, J.; Perazzo, Y.; Colmenárez, V.; López, J. Equine Pythiosis: Report in Crossed Bred (Criole Venezuelan) Horses. *Mycopathologia* **2012**, *174*, 511–517.
238. Pérez, R.C.; Luis-León, J.J.; Vivas, J.L.; Mendoza, L. Epizootic Cutaneous Pythiosis in Beef Calves. *Vet. Microbiol.* **2005**, *109*, 121–128.
239. Mendoza, L.; Arias, M.; Colmenarez, V.; Perazzo, Y. Intestinal Canine Pythiosis in Venezuela Confirmed by Serological and Sequencing Analysis. *Mycopathologia* **2005**, *159*, 219–222.
240. Márquez, A.; Salas, Y.; Canelón, J.; Perazzo, Y.; Colmenárez, V.; et al Anatomopathological Description of Equine Skin Pythiosis. *Rev. Fac. Cienc. Vet. Univ. Cent. Venezuela* **2010**, *51*, 37–42.
241. Luis-León, J.; Pérez, R.; Vivas, J.; Mendoza, L.; Alonso, F. Confirmación de Pythium Insidiosum Como Agente Etiológico de La Granulomatosis Enzoótica Bovina Mediante Análisis de Secuencia. *Salus Online* **2009**, *12*, 205–215.
242. Neufeld, A.; Seamone, C.; Maleki, B.; Heathcote, J.G. Pythium Insidiosum Keratitis: A Pictorial Essay of Natural History. *Can. J. Ophthalmol.* **2018**, *53*, e48–e50.
243. Mendoza, L.; Villalobos, J.; Calleja, C.E.; Solis, A. Evaluation of Two Vaccines for the Treatment of Pythiosis Insidiosi in Horses. *Mycopathologia* **1992**, *119*, 89–95.
244. Alfaro, A.; Mendoza, L. Four Cases of Equine Bone Lesions Caused by Pythium Insidiosum. *Equine Vet. J.* **1990**, *22*, 295–297.
245. Mendoza, L.; Alfaro, A.; Villalobos, J. Bone Lesions Caused by Pythium Insidiosum in a Horse. *Sabouraudia* **1988**, *26*, 5–12.
246. Hung, C.; Leddin, D. Keratitis Caused by Pythium Insidiosum in an Immunosuppressed Patient With Crohn's Disease. *Clin. Gastroenterol. Hepatol.* **2014**, *12*, A21–A22.
247. Mendoza, L.; Alfaro, A.A. Equine Pythiosis in Costa Rica: Report of 39 Cases. *Mycopathologia* **1986**, *94*, 123–129.
248. Wang, X.; Lu, X.; Li, S.; Wang, T.; Jia, Y.; Wang, S.; Wang, J.; Dong, C.; Shi, W. Clinical Manifestations and Treatment Outcomes of Rare Genera Fungal Keratitis in China. *Res. Sq. Prepr.* **2020**, doi:10.21203/rs.2.14305/v2.

249. He, H.; Liu, H.; Chen, X.; Wu, J.; He, M.; Zhong, X. Diagnosis and Treatment of Pythium Insidiosum Corneal Ulcer in a Chinese Child: A Case Report and Literature Review. *Am. J. Case Rep.* **2016**, *17*, 982–988.
250. Zhang, X.; Qi, X.; Lu, X.; Gao, H. Clinical Features and Treatment Prognoses of Pythium Keratitis. *Zhonghua Yan Ke Za Zhi Chin. J. Ophthalmol.* **2021**, *57*, 589–594.
251. Hou, H.; Wang, Y.; Tian, L.; Wang, F.; Sun, Z.; Chen, Z. Pythium Insidiosum Keratitis Reported in China, Raising the Alertness to This Fungus-like Infection: A Case Series. *J. Med. Case Reports* **2021**, *15*, 1–7.
252. Romero, A.; García, J.; Balestié, S.; Malfatto, F.; Vicentino, A.; Sallis, E.S.V.; Schild, A.L.; Dutra, F. Equine Pythiosis in the Eastern Wetlands of Uruguay. *Pesqui. Veterinária Bras.* **2019**, *39*, 469–475.
253. Schanzembach, M.; Brayer, D.; Sallis, S.; César, D.; Matto, C.; Almeida, R.; Nan, F.; Rodríguez, V.; Parodi, P.; Pereira, M.; et al. Descripción de Un Caso de Pitiosis Cutánea Equina y Su Diagnóstico Mediante Diversas Técnicas. *Vet. Montev.* **2019**, *55*, 96–101.
254. Hernandorena Bentancor, M.E.; Jackson Osaba, E. Reporte de Un Caso de Pitiosis Cutánea En Un Equino, Universidad de la Republica: Montevideo Uruguay, 2018.
255. Austwick, P.; Copland, J. Swamp Cancer. *Nature* **1974**, *250*, 84–84.
256. Maeno, S.; Oie, Y.; Sunada, A.; Tanibuchi, H.; Hagiwara, S.; Makimura, K.; Nishida, K. Successful Medical Management of Pythium Insidiosum Keratitis Using a Combination of Minocycline, Linezolid, and Chloramphenicol. *Am. J. Ophthalmol. Case Rep.* **2019**, *15*, 100498.
257. Ichitani, T.; Amemiya, J. Pythium Gracile Isolated from the Foci of Granular Dermatitis in the Horse (Equus Caballus). *Trans. Mycol. Soc. Jpn. Jpn.* **1980**, *21*, 263–265.
258. Barequet, I.S.; Lavinsky, F.; Rosner, M. Long-Term Follow-up after Successful Treatment of Pythium Insidiosum Keratitis in Israel. In Proceedings of the Seminars in Ophthalmology; Taylor & Francis, 2013; Vol. 28, pp. 247–250.
259. Tanhehco, T.Y.; Stacy, R.C.; Mendoza, L.; Durand, M.L.; Jakobiec, F.A.; Colby, K.A. Pythium Insidiosum Keratitis in Israel. *Eye Contact Lens* **2011**, *37*, 96–98.
260. Bernheim, D.; Dupont, D.; Aptel, F.; Dard, C.; Chiquet, C.; Normand, A.C.; Piarroux, R.; Cornet, M.; Maubon, D. Pythiosis: Case Report Leading to New Features in Clinical and Diagnostic Management of This Fungal-like Infection. *Int. J. Infect. Dis.* **2019**, *86*, 40–43.
261. Del Castillo-Jiménez, M.; Baptista-Diaz, N.; Montero, J.; Pascual, A. Pythium Insidiosum Ocular Infection. *Enferm. Infecc. Microbiol. Clin.* **2013**, *31*, 118–119.
262. Franco, D.M.; Aronson, J.F.; Hawkins, H.K.; Gallagher, J.J.; Mendoza, L.; McGinnis, M.R.; Williams-Bouyer, N. Systemic Pythium Insidiosum in a Pediatric Burn Patient. *Burns* **2010**, *36*, e68–e71.
263. Estrada-Coates, A.; Alva-Trujillo, M.; Muñoz-Melgarejo, S.; Canales-Rubio, M.; Herrera-Camacho, J. Surgical Management and Immunotherapy of Pythium Insidiosum in a Horse: Case Report. *Abanico Veterinario* **2021**, *11*, 1–10.
264. Badenoch, P.R.; Coster, D.J.; Wetherall, B.L.; Brettig, H.T.; Rozenbilds, M.A.; Drenth, A.; Wagels, G. Pythium Insidiosum Keratitis Confirmed by DNA Sequence Analysis. *Br. J. Ophthalmol.* **2001**, *85*, 502–503.
265. Sohn, Y.S.; Kim, D.Y.; Kweon, O.K.; Seo, I.B. Enteric Pythiosis in a Jindo Dog. *Korean J. Vet. Res.* **1996**, *36*, 447–451.
266. Pan, J.H.; Kerkar, S.P.; Siegenthaler, M.P.; Hughes, M.; Pandalai, P.K. A Complicated Case of Vascular Pythium Insidiosum Infection Treated with Limb-Sparing Surgery. *Int. J. Surg. Case Rep.* **2014**, *5*, 677–680.
267. Virgile, R.; Perry, H.D.; Pardanani, B.; Szabo, K.; Rahn, E.K.; Stone, J.; Salkin, I.; Dixon, D.M. Human Infectious Corneal Ulcer Caused by Pythium Insidiosum. *Cornea* **1993**, *12*, 81–83.
268. Rivierre, C.; Laprie, C.; Guiard-Marigny, O.; Bergeaud, P.; Berthelemy, M.; Guillot, J. Pythiosis in Africa. *Emerg. Infect. Dis.* **2005**, *11*, 479–481.

269. Murdoch, D.; Parr, D. Pythium Insidiosum Keratitis. *Aust. N. Z. J. Ophthalmol.* **1997**, *25*, 177–179.
270. Ros Castellar, F.; Jiménez, C.S.; del Hierro Zarzuelo, A.; Ambrosio, A.H.; de Ios Bueis, A.B. Intraocular Minocycline for the Treatment of Ocular Pythiosis. *Am. J. Health. Syst. Pharm.* **2017**, *74*, 821–825.
